# Responses of Barley to High Ambient Temperature Are Modulated by Vernalization

**DOI:** 10.3389/fpls.2021.776982

**Published:** 2022-01-25

**Authors:** Helga Ochagavía, Tibor Kiss, Ildikó Karsai, Ana M. Casas, Ernesto Igartua

**Affiliations:** ^1^Aula Dei Experimental Station (EEAD-CSIC), Zaragoza, Spain; ^2^Agricultural Institute, Centre for Agricultural Research, ELKH, Martonvásár, Hungary; ^3^Center for Research and Development, Food and Wine Center of Excellence, Eszterházy Károly Catholic University, Eger, Hungary

**Keywords:** barley, development, flowering time, *PPD-H1*, temperature, *VRN-H1*, *VRN-H2*, vernalization

## Abstract

Ambient temperatures are increasing due to climate change. Cereal crops development and production will be affected consequently. Flowering time is a key factor for adaptation of small grain cereals and, therefore, exploring developmental responses of barley to rising temperatures is required. In this work, we studied phasic growth, and inflorescence traits related to yield, in eight near isogenic lines of barley (*Hordeum vulgare* L.) differing at the *VRN-H1*, *VRN-H2* and *PPD-H1* genes, representing different growth habits. The lines were grown in contrasting vernalization treatments, under two temperature regimes (18 and 25°C), in long days. Lines with recessive *ppd-H1* presented delayed development compared to lines with the sensitive *PPD-H1* allele, across the two growth phases considered. High temperature delayed flowering in all unvernalized plants, and in vernalized spring barleys carrying the insensitive *ppd-H1* allele, whilst it accelerated flowering in spring barleys with the sensitive *PPD-H1* allele. This finding evidenced an interaction between *PPD-H1*, temperature and vernalization. At the high temperature, *PPD-H1* lines in spring backgrounds (*VRN-H1-7*) yielded more, whereas lines with *ppd-H1* were best in *vrn-H1* background. Our study revealed new information that will support breeding high-yielding cultivars with specific combinations of major adaptation genes tailored to future climatic conditions.

## Introduction

Achieving a successful crop relies on a delicate balance between the use of available resources and avoidance of risks, resulting in a maximized economic output. For cereals, this output is grain yield, produced in inflorescences at the end of their annual cycle. Risks (frost, heat, pests, diseases, etc.) are not equally probable throughout the growth cycle of the plant, and neither is the availability of water and nutrients. Likewise, the plant is not equally susceptible to risks, nor is it equally sensitive to lack of resources at each growth phase. For a cereal like barley (*Hordeum vulgare* L.), flowering time is the key factor for adaptation and can be partitioned into two major developmental phases, in which different yield components are determined: (i) time from sowing to appearance of first stem node (containing vegetative and early reproductive phases), in which leaves and spikelets are formed, and (ii) late reproductive (Slafer and Rawson, 1994), in which floret development takes place. The morphological milestones plant emergence, appearance of the first node, and heading time, set the limits for these phases. Barley phasic development is controlled by an interaction of genetic and environmental factors. The main environmental factors are photoperiod and temperature (Laurie et al., 2004). On the other hand, allelic combinations at key loci determine the onset and duration of developmental phases, affecting plant resource allocation and source-sink balance. Adaptation to a specific environment is achieved by selection of the best combinations of alleles that optimize yield at each specific environment.

Barley (like wheat) can be sown in autumn or spring. Barleys adapted to autumn sowing are said to have a “winter growth habit,” whereas those sown in spring display a “spring growth habit,” the growth habit corresponding to the set of genetic features making the crop particularly fitting to each sowing date. Winter type-barleys require a period of low temperatures (vernalization) to accelerate flowering (while contributing to withstand frosts), whilst spring barleys grow in absence of cold temperatures (Trevaskis et al., 2003).

Although barley is a crop exhibiting one of the widest distributions across agroecological zones, current adaptation syndromes may not be optimum for some of the new conditions generated by climate change, posing an extra burden on plant breeders. New cultivars should be prepared to withstand and even thrive in the new climatic conditions. Under these circumstances, it seems sensible to explore and test new allelic combinations (or at least new to a particular region) of the key development genes that may perform better than the current ones.

Among the changes expected in climate, there is wide consensus on the increase of daily and night ambient temperatures (IPCC, 2013). This increase may affect barley growth in different ways. On the one hand, it may compromise fulfilling the period of cold temperature required by winter-type barleys to complete vernalization. On the other hand, higher ambient temperatures are predicted to hasten growth (Asseng et al., 2015) and may affect gamete production and spike fertility (Prasad and Djanaguiraman, 2014). It has been reported that warmer night temperatures during the critical period, comprising from the third detectable node to 10 days after anthesis, caused a shorter grain filling with negative consequences on grain yield, associated to decrease of grain number (García et al., 2015, 2016; Giménez et al., 2021). It is crucial to explore crop developmental responses to climate change. Particularly, it is important to understand how elevated temperature affects flowering time and its components: (i) duration of pre-flowering phases, (ii) number of leaves initiated in main shoots and their rate of appearance (or its inverse, phyllochron, defined as thermal time between the emergence of two successive leaves) (Slafer and Rawson, 1994; Jamieson et al., 1998). Likewise, it is essential to analyze how genetic variation in genes regulating flowering time influences or modifies the responses to high temperatures (Kiss et al., 2017).

Vernalization requirement in barley is controlled by three loci: *VRN-H1*, *VRN-H2* and *VRN-H3*, located on chromosomes 5H, 4H and 7H, respectively. *VRN-H1* is a flowering inducer gene that encodes an *AP1*-like MADS-box transcription factor (Danyluk et al., 2003; Trevaskis et al., 2003; Yan et al., 2003). *VRN-H2* is a flowering repressor, encoded by a zinc finger-CCT (*CONSTANS*, *CONSTANS-like*, and *TOC*) domain transcription factor (*ZCCT*) (Yan et al., 2004), which is induced by long days (Karsai et al., 2005; Dubcovsky et al., 2006; Trevaskis et al., 2006). Winter barleys combine a recessive allele at *VRN-H1* and the presence of *VRN-H2*. In these genotypes, *VRN-H1* is repressed by lack of cold. After a certain time under cold temperatures has passed, its expression induces the repression of *VRN-H2*, allowing promotion to flowering (Yan et al., 2004; Trevaskis et al., 2006; Oliver et al., 2013). On the other hand, dominant *VRN-H1* alleles, carried by spring genotypes, present a constitutive expression, even without vernalization (Hemming et al., 2009). Plants with a recessive *VRN-H1* and lacking *VRN-H2* are described as having a facultative growth habit (Muñoz-Amatriaín et al., 2020). They present a minor vernalization response, and can be sown either in autumn or spring. *VRN-H3* is the barley ortholog of the Arabidopsis *FLOWERING LOCUS T* gene (Yan et al., 2006). *VRN-H3* integrates the flowering signals from the photoperiod and vernalization pathways (Kikuchi et al., 2009).

Two main photoperiod response genes in barley, *PPD-H1* and *PPD-H2* map to chromosomes 2H and 1H, respectively. *PPD-H1* (*HvPRR37*) regulates flowering under long days (Turner et al., 2005; Campoli et al., 2012) and *PPD-H2* (*HvFT3*) under short days (Faure et al., 2007; Kikuchi et al., 2009). The ancestral, dominant *PPD-H1* allele accelerates flowering in long days (LDs), and is the most frequent in winter barleys. A natural mutation in the CCT domain of the *PPD-H1* locus is associated with late flowering time under LDs (Turner et al., 2005), and is prevalent in spring barleys.

Furthermore, an interaction between high ambient temperatures and photoperiod has been reported (Hemming et al., 2012). Ejaz and von Korff (2017) showed that, at high temperatures (28°/24°C day/night), lines carrying the sensitive *PPD-H1* allele, in conjunction with a spring *VRN-H1* allele, accelerated plant development, whereas it was delayed in the presence of insensitive *ppd-H1* and winter *vrn-H1* allele. These findings suggest that *PPD-H1* and *VRN-H1* interact to control the development under high temperatures.

In this study, we aim to analyze the combined effects of diverse environmental conditions, namely optimum or high temperatures and presence or absence of vernalization, in a set of barley isolines with different alleles of *VRN-H1*, *VRN-H2* and *PPD-H1* on development, yield components and gene expression.

## Materials and Methods

### Plant Material and Growth Conditions

Eight near isogenic lines (NILs) were used in this study. These lines were developed at CSIRO Agriculture and Food (Canberra, Australia), after five rounds of crossing, up to BC_4_, using different donors of *VRN-H1*, *VRN-H2* and *PPD-H1* alleles into the facultative barley cultivar “WI4441” (Oliver et al., 2013). Additionally, two controls were grown, Dicktoo and Kompolti korai (Karsai et al., 2001, 2008). Dicktoo is a facultative genotype, while Kompolti korai has winter growth habit. Both of them are photoperiod-sensitive genotypes. Photoperiod-sensitive and insensitivity barleys exhibit a quantitative response to photoperiod. All barleys accelerate the development under long photoperiods, but not at the same rate. Under long days, photoperiod–sensitive genotypes accelerate the rate of development more than photoperiod-insensitive genotypes. More details about the lines are shown in Table 1 and Supplementary Figure 1.

**TABLE 1:** Genetic constitution of the barley genotypes analyzed in this study, at *VRN-H1*, *VRN-H2*, *PPD-H1* and *PHYC*, and growth habit.

Genotype	VRN-H1	VRN-H2	PPD-H1	PHYC	Growth habit
C01	*vrn-H1*	*VRN-H2*	*PPD-H1*	*PHYC-e*	Winter, LD sensitive
C02	*vrn-H1*	*VRN-H2*	*ppd-H1*	*PHYC-e*	Winter, LD-insensitive
C03	*vrn-H1*	*vrn-H2*	*PPD-H1*	*PHYC-e*	Facultative, LD sensitive
C04	*vrn-H1*	*vrn-H2*	*ppd-H1*	*PHYC-e*	Facultative, LD insensitive
C05	*VRN-H1-7*	*VRN-H2*	*PPD-H1*	*PHYC-l*	Spring, LD sensitive
C06	*VRN-H1-7*	*VRN-H2*	*ppd-H1*	*PHYC-l*	Spring, LD insensitive
C07	*VRN-H1-7*	*vrn-H2*	*PPD-H1*	*PHYC-l*	Spring, LD sensitive
C08	*VRN-H1-7*	*vrn-H2*	*ppd-H1*	*PHYC-l*	Spring, LD insensitive
Dicktoo	*vrn-H1*	*vrn-H2*	*PPD-H1*	*PHYC-l*	Facultative, LD sensitive
Kompolti korai	*vrn-H1*	*VRN-H2*	*PPD-H1*	*PHYC-l*	Winter, LD sensitive

The experiment was carried out in the Phytotron facilities of the Agricultural Research Institute, Hungarian Academy of Sciences, Martonvásár, using CONVIRON PGR-15 growth chambers (Conviron Ltd., Canada). Plants were grown in pots (one plant per pot), 12 cm in diameter and 18 cm in height, with a soil capacity of 1.5 kg filled with a 4:1 mixture of garden soil and sand.

The study consisted of the factorial combination of two vernalization treatments (vernalized or unvernalized) and two temperature regimes (constant 18°C or 25°C). Seeds were planted in jiffy pots. After emergence, the plants were moved to the vernalization chamber for 60 days, at 3–4°C, under 8 h photoperiod and low light intensity [12–13 μmol m^–2^ s^–1^ photosynthetic photon flux density (PPFD)]. When the vernalization treatment was completed, plants were transplanted into individual pots and transferred to two growth chambers set at constant temperatures of either 18°C (close to the optimum level) or 25°C (above the optimum temperature level), both under 16 h of light and an intensity of 240 μmol m^–2^ s^–1^ PPFD, provided by Tungsram HGL-400 metal halide bulbs. Seeds for the non-vernalized treatments were germinated in jiffy pots, at room temperature, 7 days prior to the start of the experiment, and were transplanted to individual pots at the same time as the vernalized plants. In order to match starting points of development, a correction was performed, as all unvernalized plants were consistently slightly less developed than their counterparts in the vernalized treatments. The correction considered the difference in the initial number of leaves of the vernalized and unvernalized treatments. Through the analysis of dynamics of leaves of unvernalized treatments, we estimated the thermal time at which the number of leaves matched that of the vernalized treatment, for each genotype and temperature. These values of thermal time were subtracted from the developmental variables recorded at the unvernalized treatment. In this way, plants from both vernalization treatments started the cycle with the same number of leaves. Developmental phases Z31 (first node detectable, starting of stem elongation), Z31-49, and Z49 (awn appearance), determined according to the decimal code developed by Zadoks et al. (1974), were corrected with this method in both unvernalized treatments. Four replicates (individual plants) per genotype and treatment were used.

### Measurements

Besides phenological stages Z31 and Z49, number of leaves per main stem were recorded twice per week in four plants per genotype and treatment (Haun, 1973). With these records, the dynamics of leaf appearance per genotype were determined. The number of leaves monitored in each treatment was plotted against thermal time. Sequential number of leaves for each genotype were fitted by linear regression to calculate phyllochron.

At maturity, grain yield, number of spikelets per main spike, number of grains per spikelet and per main spike, and thousand grain weight were measured in the main shoots of four plants per genotype and treatment.

### Statistical Analysis

Analyses of variance (ANOVA) were performed for all the traits within each treatment using GenStat (18th Edition, VSN International, Hemel Hempstead, United Kingdom). The ANOVA models included temperature, vernalization, and genotypes, as fixed factors, and all their possible interactions, and replicates nested within temperature and vernalization treatments. The factor genotype was further divided into the three genes, *VRN-H1, VRN-H2, PPD-H1*, and their interactions. Several models were run, differing in the set of near isogenic lines considered in each one. Since C01 and C02 did not reach Z31 nor Z49 by the end of the experiment when not vernalized, the first model included the eight NILs, with missing values for C01 and C02 in unvernalized treatments (Table 2). The end date of the experiment was given as proxy to facilitate carrying out a statistical analysis with the full set of data (estimation for 18°C 1,800°Cd, and for 25°C 2,500°Cd, respectively). The second model also involved the eight NILs, but including the estimated values of Z31 and Z49 for unvernalized C01 and C02, Supplementary Table 1). The third model was restricted to NILs C03 to C08 (Supplementary Table 2), whilst the fourth was restricted to NILs C05 to C08 (Supplementary Table 3).

**TABLE 2:** Mean squares from the analyses of variance for the main developmental traits, considering only the eight NILs.

Source of variation	df	Z49 (°C d)	Z31 (°C d)	Z31-49 (°C d)	FLN (#leaves)	Phyllochron (°C d)

**Temperature (T)**	**1**	185,157 ***	9,242 ***	114,698 ***	1.9 ***	4,909 ***
**Vernalization (V)**	**1**	**1,268 ns**	11,945 ***	7,292 *	0.5**	**33 ns**
**T.V**	**1**	113,543 ***	28,371 ***	29,940 ***	3.9 ***	673 ***
**Residual 1**	**12**	**1,257**	**131**	**1,186**	**0.1**	**10**
**Genotype**	**7**	620,018 ***	97,618 ***	273,992 ***	14.6 ***	4,165 ***
*VRN-H1*	1	664,715 ***	874 *	635,925 ***	5.3 ***	4,126 ***
*VRN-H2*	1	692 ns	3,521 ***	2,001 ns	0.1 ns	101 ***
*PPD-H1*	1	3,383,990 ***	582,798 ***	1,117,033 ***	79.5 ***	20,590 ***
*VRN-H1*.*VRN-H2*	1	67,929 ***	43,823 ***	1,567 ns	4.5 ***	323 ***
*VRN-H1*. *PPD-H1*	1	197,793 ***	4,764 ***	156,001 ***	5.7 ***	3,336 ***
*VRN-H2*. *PPD-H1*	1	24,786 ***	46,758 ***	1,564 ns	0.7 ***	160 ***
*VRN-H1*.*VRN-H2*.*PPD-H1*	1	220 ns	784 *	3,856 *	6.6 ***	522 ***
Genotype*Temperature	**7**	28,976 ***	13,121 ***	15,099 ***	0.2 ***	431 ***
*VRN-H1*.T	1	19,733 ***	2,380 ***	7,601 *	0.1 ns	181 ***
*VRN-H2*.T	1	7,629 ***	5,445 ***	82 ns	0.0 ns	287 ***
*PPD-H1*.T	1	172,253 ***	33,356 ***	54,033 ***	0.0 ns	2,238 ***
*VRN-H1*. *VRN-H2*.T	1	3 ns	20,283 ***	21,105 ***	0.0 ns	195 ***
*VRN-H1*.*PPD-H1*.T	1	116 ns	47 ns	15 ns	1.2 ***	195 ***
*VRN-H2.PPD-H1*.T	1	1,572 ns	11,953 **	5,511 **	0.0 ns	3 ns
*VRN-H1*.*VRN-H2*.*PPD-H1*.T	1	1,527 ns	29,143 ***	17,342 ***	0.0 ns	29 *
Genotype*Vernalization	**5**	37,181 ***	33,094***	17,345 ***	0.2 ***	863 ***
*VRN-H1*.V	1	91,374 ***	46,041 ***	5,777 **	0.5 **	2,970 ***
*VRN-H2*.V	1	10,338 ***	6,479 ***	1,082 ns	0.0 ns	27 *
*PPD-H1*.V	1	1,328 ns	76,197 ***	67,004 ***	0.1 ns	63 **
*VRN-H1*.*VRN-H2*.V	0					
*VRN-H1*.*PPD-H1*.V	1	73,937 ***	25,569 ***	8,604 ***	0.1 ns	1,108 ***
*VRN-H2*. *PPD-H1*.V	1	280 ns	2,182 **	115 ns	0.4 **	15 ns
*VRN-H1*.*VRN-H2*. *PPD-H1*.V	0					
Genotype*Temperature* **Vernalization**	**5**	20,034 ***	12,893 ***	18,597 ***	1.2 ***	160 ***
*VRN-H1*.T.V	1	10 ns	5,176 ***	5,403 **	0.2 *	125 ***
*VRN-H2*.T.V	1	10,157 ***	18,838 ***	1,679 ns	0.0 ns	139 ***
*PPD-H1*.T.V	1	41,169 ***	27,622 ***	1,351 ns	5.1 ***	36 *
*VRN-H1*.*VRN-H2*.T.V	0					
*VRN-H1*.*PPD-H1*.T.V	1	43,880 ***	1,054 *	58,562 ***	0.9 **	424 ***
*VRN-H2*. *PPD-H1*.T.V	1	1,628 ns	11,414 ***	21,681 ***	0.0 ns	1 ns
*VRN-H1*.*VRN-H2*. *PPD-H1*.T.V	0					
**Residual 2**	**72**	**605**	**169**	**712**	**0.1**	**6**

Z49: time until awns just visible above the last leaf sheath (flowering time), Z31: time until first node appearance at the base of the main stem, Z31-49: duration of late reproductive phase, FLN: final leaf number. ns, *, **, ***, indicates non-significant, and significance at the 0.05, 0.01, and 0.001 probability level, respectively. C01 and C02, unvernalized treatments, set as missing values. In bold, values of the main factors of the analyses.

A correlation network analysis was carried out with the R package “qgraph” (Epskamp et al., 2012). A principal multiple factorial analysis (MFA) was performed using the R package FactoMineR (Lê et al., 2008). The R package Factoextra (Kassambara and Mundt, 2020) was employed for extracting and visualizing the results. The MFA summarizes the observations described by a set of variables structured into three groups (Phenology-development, Yield, Gene expression). These groups gather the quantitative variables measured in each experiment. Each variable within a group was equally weighted, so the influence of each set of variables in the analysis was balanced.

### Genotyping

Genomic DNA was extracted from ground frozen leaves of individual plants with the EchoLUTION Plant DNA kit (BioEcho Life Sciences, GmbH). Genotyping was carried out first with diagnostic markers for the main flowering time genes, using gene specific primers for *VRN-H1*, *VRN-H2* and *PPD-H1* as reported (Turner et al., 2005; von Zitzewitz et al., 2005). Other genes analyzed were *PPD-H2* (candidate gene for *HvFT3*), *HvCEN* and *VRN-H3*, following previously published protocols (Mansour et al., 2018). Variation in *PHYC* was assessed using a diagnostic KASP marker for the T/C SNP in exon 1 as described by Hill et al. (2019). All near isogenic lines used in this study have dominant alleles of *PPD-H2*, haplotype II of *HvCEN*, and the combination of late promoter, and SNPs TC (early) in the first intron of *VRN-H3*. Besides, whole genome genotyping of the 8 NILs was performed with the 50k Illumina SNP chip (Bayer et al., 2017), as shown in Supplementary Figure 1.

During seed multiplication, lines C01 to C04 flowered surprisingly earlier than lines C05-08, even after being vernalized (Supplementary Figure 2). Since it is known that *VRN-H1* is closely linked to *PHYC*, a known source of earliness in barley (Nishida et al., 2013; Pankin et al., 2014), we genotyped *PHYC* in the NILs with a diagnostic marker. It turned out that lines C01-C04 carry the *PHYC-early* allele (C, from here on, *PHYC-e*, derived from Haruna Nijo in the WI441 genetic background, Laws et al., 2010), linked to the wild-type winter *vrn-H1* allele, whereas C05-C08 have the *PHYC*-*late* allele (T, from here on referred to as *PHYC-l*), linked to the introgressed *VRN-H1-7* spring allele. Linkage between *VRN-H1* and *PHYC* prevents full separation of the effects of these genes in this study but, on the other hand, allows gaining insight on the performance of the latter when subjected to variable environmental conditions.

### Gene Expression

For RNA extraction, leaf tissue was harvested at 0, 250 and 350°C d from the onset of temperature treatments. Samples were collected from the last fully expanded leaf, at the middle of the day (8 h after lights were turned on), immediately frozen in liquid nitrogen and stored at −80°C. Total RNA was isolated using Trizol Reagent (Thermo Fisher Scientific, Ltd.) and the Qiagen RNeasy Plant Mini Kit (Qiagen, Hilden, Germany), following the manufacturer instructions. Synthesis of cDNA was performed with 1 μg of total RNA using the RevertAid First Strand cDNA synthesis kit (Thermo Scientific Ltd.), with the standard protocol provided by the company. qRT-PCR was carried out for three biological, and two technical replicates, in a Rotor-Gene Q equipment (Qiagen, Hilden, Germany) using the SYBR-Green technology and the corresponding standard protocol provided by the company. Expression was calculated using the Rotor-Gene software, which also considers the amplification efficiency. Relative expression was normalized against the geometric mean of two housekeeping genes, *Actin* and *DCP5*, according to Vandesompele et al. (2002). To test whether *VRN-H2* expression occurred at any point during the light period, NILs C05 and C06 were grown under long photoperiod (16 h light/8 h dark) and constant temperatures of 18 and 25°C without prior vernalization, in an independent experiment. Leaf samples, three biological replicates, were sequentially collected every 3 h, starting from 3.5 to 15.5 h of light, for a total of five data points, 13 days after sowing. RNA was extracted using the Total RNA Mini Kit (IBI Scientific), and quantified using a NanoDrop 2000 (Thermo Fisher Scientific). The synthesis of first-strand cDNA was carried out with SuperScript III (Invitrogen) and derived from 1 μg RNA. Primer sequences used for qRT-PCR are showed in Supplementary Table 4.

## Results

### Development

**FIGURE 1:**
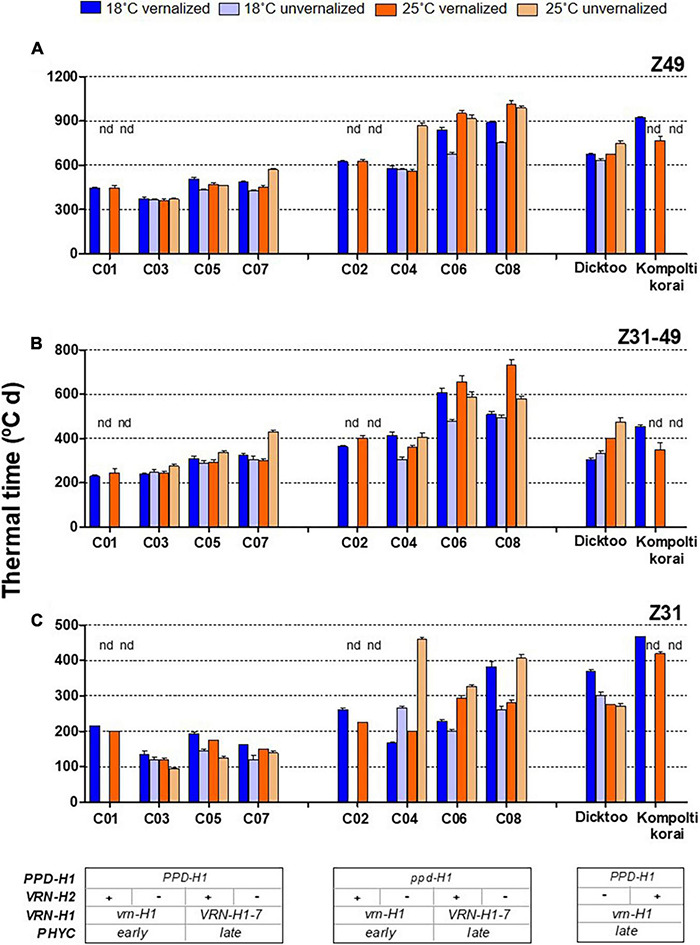
Plant growth duration from the onset of the experiment until flowering **(A)** from onset of stem elongation until flowering time **(B)** and from the onset of experiment until the appearance of the first node **(C)** for different barley NILs, whose genetic background is indicated at the bottom of the figure (inset tables). The four treatments, combinations of vernalization or absence of vernalization and temperature (18°C or 25°C) are color coded. Lines C01, C03, C05 and C07 carry dominant *PPD-H1* alleles, whilst C02, C04, C06 and C08 carry a recessive *ppd-H1*. “nd” means not-determined. Dicktoo and Kompolti korai are check cultivars. Bars represent mean ± standard error of the mean (SE).

Temperature, vernalization, and genotype had great impact on development. There were large and significant differences in thermal time until flowering (Z49) and its component phases (Z31 and Z31-49), due to these three factors (Figure 1, Table 2, and Supplementary Figure 3).

Regarding growth habit, isogenic lines C01 and C02 are winter types, C03 and C04 are facultative (winter allele at *VRN-H1* and absence of *VRN-H2*), and lines C05-C08 are spring types. The patterns of development for the winter barleys essentially followed the expectations based on growth habit. Lines C01, C02, and Kompolti korai did not reach Z31 without vernalization, as their combination of winter alleles at *VRN-H1* and *VRN-H2* elicits a strong and compulsory vernalization response (Figure 1 and Supplementary Figure 4). We assigned C01 and C02 estimated Z31 and Z49 values of 1,800 and 2,500°C d, at 18°C and 25°C unvernalized treatments, respectively, as they correspond to the date of termination of the experiment. The results of the analyses including these estimates are presented in Supplementary Table 1. These estimates were not used in the analyses included in the main text, unless stated otherwise.

The large variation detected for flowering time, its component phases, final leaf number (FLN) and phyllochron was dominated by differences between genotypes, and to a lesser extent, though still highly significant, by temperature and interactions of genotype with both temperature and vernalization (Table 2). The only exception was phyllochron, whose variation was slightly more affected by temperature than by genotypes.

**FIGURE 2:**
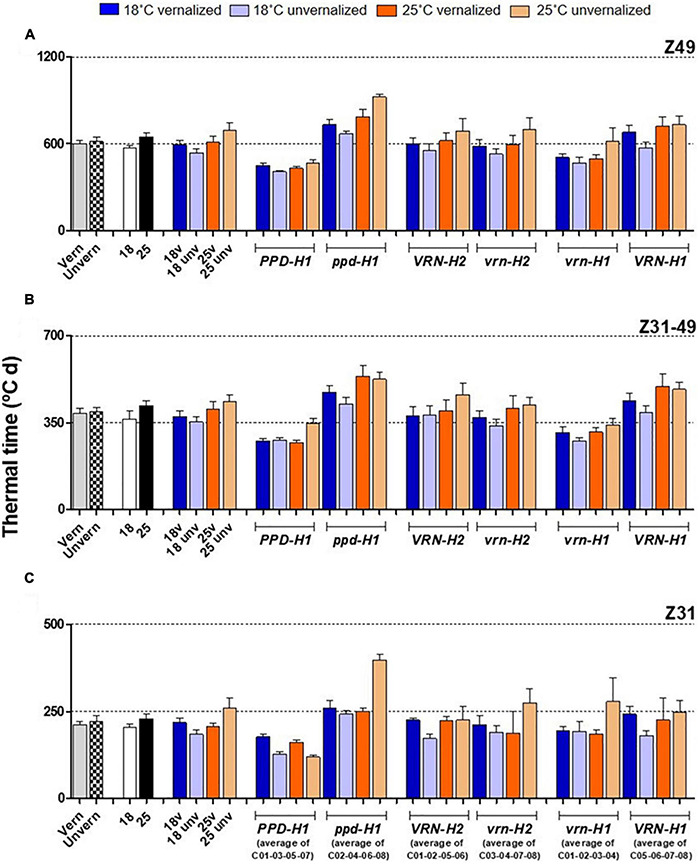
Duration of plant growth phases, with genotypes averaged across classes. The first 8 bars on the left of each panel represent averages of the 8 barley NILs, split by treatments: vernalized and unvernalized, 18°C and 25°C, and the four treatments combining vernalization and temperature. The other bars represent averages of the 4 NILs carrying particular alleles at the three genes polymorphic in these NILs, as indicated in the legend of the X-axes: lines classified by *PPD-H1* alleles; lines classified by *VRN-H2* alleles, and lines classified by *VRN-H1* alleles. Duration since the onset of the experiment until flowering **(A)** from onset of stem elongation until flowering **(B)** from the onset of the experiment to the appearance of the first node **(C)**. Bars represent mean ± standard error of the mean (SE). Means were calculated without unvernalized C01 and C02 lines.

The effect of vernalization (V) was remarkable on the phase until Z31, and small afterward, with null effect on the total duration until Z49 (Table 2 and Supplementary Figure 3). At 18°C, genotypes responded differently to vernalization: time to Z49 was almost unchanged in facultative isolines (C03, C04), whereas spring lines C05-C08 and check Dicktoo suffered a slight delay due to vernalization (Figure 1A). However, at 25°C, there were few differences due to vernalization except in lines C04 and C07. These observations evidenced a significant temperature × vernalization × genotype interaction (Table 2 and Figure 1A). Overall, temperature (T) induced large variations on flowering time and its two phases (Table 2 and Figure 2). High temperature consistently increased the length of phase Z31-49, but it increased the length of the phase until Z31 only for unvernalized plants (Figure 2). The delay in flowering time caused by temperature was not consistent across vernalization and genotypes, as indicated by the large interactions found (Table 2, Supplementary Tables 1, 3, and Supplementary Figure 3). There was a significant V × T interaction, due to a crossover effect: vernalized plants had a longer cycle at 18°C and a shorter one at 25°C than unvernalized ones (Figure 2). Significant vernalization and vernalization-by-temperature effects on the time until Z31 were still detected in the analysis limited to purely spring lines C05 to C08. This is surprising, given that all the genotypes with any theoretical response to vernalization were excluded from this analysis (Supplementary Table 3).

**FIGURE 3:**
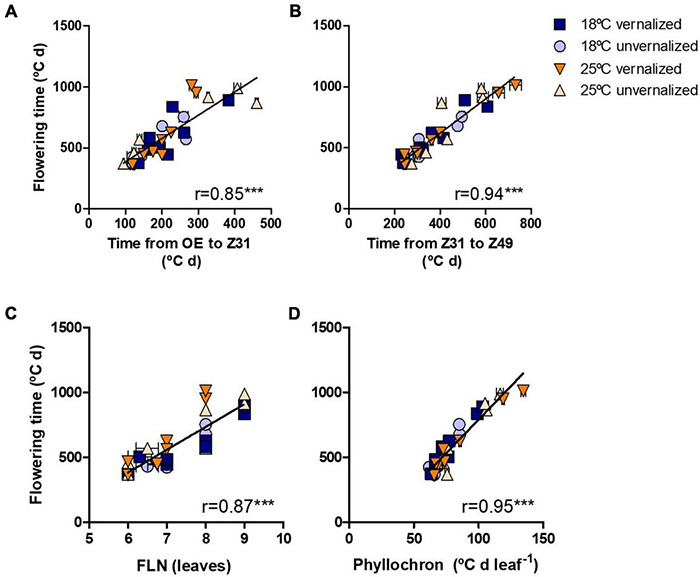
Relationship between time until flowering (Z49) and **(A)** duration from OE-onset of experiment to onset of stem elongation (Z31), **(B)** duration of the late reproductive phase (Z31-49), **(C)** final leaf number, and **(D)** phyllochron for eight NILs subjected to non-vernalized conditions at 18°C (circles) or 25°C (triangles) and those which were vernalized and then grown at 18°C (squares) or 25°C (down-triangles).

**FIGURE 4:**
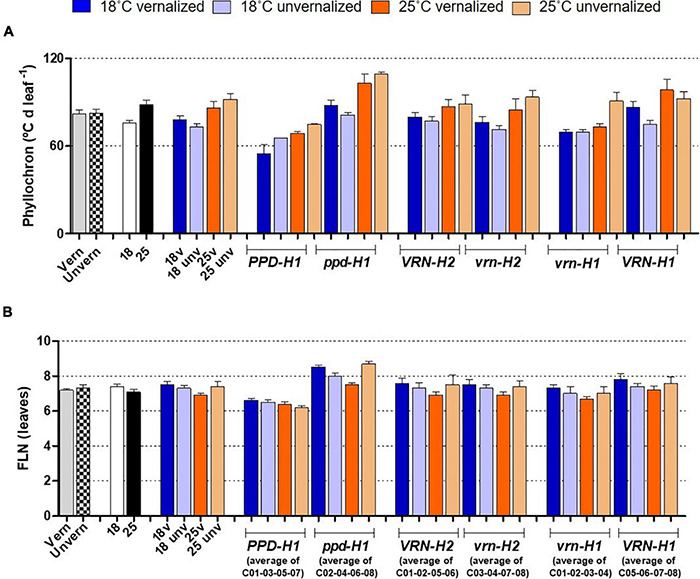
Phyllochron **(A)** and final leaf number **(B)**, with genotypes averaged across classes. The first 8 bars on the left of each panel represent averages of the 8 barley NILs, split by treatments: vernalized and unvernalized, 18°C and 25°C, and the four treatments combining vernalization and temperature. The other bars represent averages of the 4 NILs carrying particular alleles at the three genes polymorphic in these NILs, as indicated in the legend of the X-axes: lines classified by *PPD-H1* alleles; lines classified by *VRN-H2* alleles, and lines classified by *VRN-H1* alleles. Bars represent mean ± standard error of the mean (SE). Means were calculated without unvernalized C01 and C02 lines, which failed to reach Z31 stage.

Flowering time (Z49) was more related to the duration of the late reproductive phase (Z31-Z49), and to the phyllochron, than to changes in the duration of the phase until Z31, or final leaf number (Figure 3). Phyllochron was closely related to the duration of the late reproductive phase (*r* = 0.91, *P* < *0.001*, Supplementary Figure 5A). It increased remarkably at the high temperature treatment, whereas it was roughly unchanged by the vernalization factor (Figure 4A). FLN was less affected in general by the treatments. It is worth noting that vernalization reduced FLN only at 25°C, not at 18°C, producing a significant temperature by vernalization interaction (Table 2 and Figure 4B).

The effect of *PPD-H1* was the main driver of genotypic variation in this experiment, being the single most relevant factor in the analyses of variance for most traits (65, 55, 46, 65 and 47%, for flowering time, Z31, Z31-49, FLN and phyllochron, respectively), (Table 2, Supplementary Figure 3, and Figure 1). Plants carrying the insensitive *ppd-H1* displayed delayed flowering across treatments (Figures 1A, 2A), between pairs of isolines (Supplementary Figure 6). In particular, high temperature caused the highest developmental delay of these lines when not vernalized at 25°C (255°C d), an effect 5 times larger than the one observed in the same lines when vernalized (54°C d, Figure 2).

The development of plants with sensitive *PPD-H1* was almost unchanged by temperature when vernalized (452 *vs.* 431°C d on average at 18°C and 25°C, respectively). However, when not vernalized at 25°C, they suffered a slight but significant average delay of 59°C d until Z49, very similar to the average delay experienced by *ppd-H1* recessive plants at 25°C when vernalized (54°C d, Figure 2).

The allelic effect of *PPD-H1* was detected in the two stages of development, but it was larger in general on the second phase (Z31-49, Table 2 and Figure 2). However, there was a marked allelic effect in the first phase due to the delay of recessive lines when lack of vernalization was combined with high temperature (Figure 1 and Supplementary Figure 5). These fluctuations of effects underpinned the significant high order interactions of *PPD-H1* with temperature, vernalization and *VRN-H1* observed for Z49, Z31 and Z31-49 (Table 2).

The high temperature effect for the genotypes carrying the *PPD-H1* dominant allele was small, only visible at line C07 unvernalized. For the *ppd-H1* allele, the temperature effect was also very low for winter and facultative genotypes (C02 and C04) when vernalized (Figure 1). When unvernalized, line C04 suffered a remarkable delay in development, caused by a lengthened phase until Z31. However, for spring *ppd-H1* lines (C06 and C08), in the unvernalized treatments, there were large temperature effects at both developmental phases, whereas when vernalization was applied, the temperature effect was, on average, large at the Z31-49 phase, and almost negligible for Z31. In this last case, however, the effect was the average of two very different responses for the period until Z31, of the two spring lines carrying the recessive *ppd-H1* allele (C06 and C08). The reaction of *PPD-H1* alleles to temperature was complex and dependent on other factors. A general conclusion is that the dominant allele of *PPD-H1* and vernalization acted additively to buffer the effects of an increasing temperature on development.

The effect of recessive *ppd-H1* on development was a combination of larger FLN and lengthening of the phyllochron (Figure 4 and Supplementary Figure 6), modified by temperature and vernalization, and specific genotypic effects.

There was no overall effect of *VRN-H2* on Z49, but it did affect the duration until Z31 and presented significant interactions with temperature and vernalization (Table 2). In spring lines (C05-C08), the effect of *VRN-H2* depended on the *PPD-H1* allele present.

In spring lines, C05-C08, the *VRN-H2* × *PPD-H1* interaction affected total duration (Z49), but through different phases (Figure 1 and Supplementary Table 3). In general, presence of *VRN-H2* shortened the first phase (Z31) in recessive *ppd-H1* lines, and the second one (Z31-49) in *PPD-H1* dominant lines (mostly due to the 25°C unvernalized treatment). *VRN-H2*-carrying lines had shorter cycle than *VRN-H2*-absent lines (41°C d) overall, but particularly in presence of recessive *ppd-H1* (67°C d *vs.* only 16°C d in presence of dominant *PPD-H1*). However, this effect was mostly overridden at 25°C, and was independent of vernalization.

Regarding *VRN-H1*, leaving out unvernalized C01 and C02, plants with the winter *vrn-H1* allele flowered earlier than those carrying spring *VRN-H1-7* (Figure 2). This result was partly due to the presence of the *PHYC-e* allele, completely linked to the winter *vrn-H1* allele. Yet, plants C01 and C02 did not flower without vernalization, indicating that the effect of *PHYC* did not override their vernalization requirement.

The effect of *PHYC* on flowering can be indirectly estimated attending to the results of vernalized treatments. When vernalized, earliness due to the presence of the different *VRN-H1/VRN-H2* haplotypes should disappear. Any differences remaining should be the result of other genes, most likely *PHYC*. There were significant differences between lines split by the *VRN-H1* alleles (likely, *PHYC* effect) in time to reach Z49 in both vernalized treatments. At both temperatures, the effect was cumulative over the two developmental phases, but was more marked on the second one (Z31-Z49) (Figure 2).

### Inflorescence Traits

Inflorescence traits recorded in each main spike were grain yield, grain number, number of spikelets, thousand grain weight, and grain number per spikelet. All these traits were affected by temperature and vernalization (Supplementary Table 5). The lack of spikes in unvernalized lines C01 and C02 caused that vernalization, *VRN-H1* and of *VRN-H2* and their interactions were highly significant, and are the dominant sources of variation in the analysis of variance with all 8 isogenic lines, including estimates for C01 and C02 unvernalized (Supplementary Table 5). The following comments pertain to the analyses in which these large effects were removed by setting all C01 and C02 unvernalized yield and yield component scores as missing values (Table 3). The findings of this first analysis were later confirmed in analyses carried out with lines C05-C08 (all spring lines, without *VRN-H1* nor *PHYC* polymorphism, Supplementary Table 3), and with lines C03-C08 (Supplementary Table 2).

**TABLE 3:** Mean squares from the analyses of variance for inflorescence traits measured at the main spike of each plant, corresponding to the eight NILs.

Source of variation	df	Grain yield (g)	Grain #	Spikelet #	TGW (g)	Grain #. spikelet^–1^

**Temperature (T)**	**1**	10.432 ***	4,315.1 ***	454.3 ***	5,770.0 ***	8.581 ***
**Vernalization (V)**	**1**	0.268 **	**14.1 ns**	**1.0 ns**	800.9 *	0.142 *
**T.V**	**1**	**0.014 ns**	39.2 *	39.2 **	**161.1 ns**	**0.079 ns**
**Residual 1**	**12**	**0.018**	**5.9**	**3.0**	**129.5**	**0.022**
**Genotype**	**7**	0.196 ***	74.6 ***	228.4 ***	1,828.6 ***	0.211 ***
*VRN-H1*	1	0.080 *	0.1 *	154.3 ***	2,232.9 ***	0.051 ns
*VRN-H2*	1	0.123 **	0.1 **	57.0 ***	58.7 ns	0.054 ns
*PPD-H1*	1	0.016 ns	187.1 ***	1,328.1 ***	6,007.1 ***	0.131 **
*VRN-H1*.*VRN-H2*	1	0.004 ns	2.7 ns	11.0 *	27.4 ns	0.003 ns
*VRN-H1*. *PPD-H1*	1	1.055 ***	308.6 ***	1.2 ns	4,179.6 ***	1.124 ***
*VRN-H2*. *PPD-H1*	1	0.087 *	23.9 *	0.3 ns	293.3 ns	0.077*
*VRN-H1*.*VRN-H2*.*PPD-H1*	1	0.005 ns	0.0 ns	47.2 ***	1.4 ns	0.037 ns
Genotype*Temperature	**7**	0.054 **	84.6 ***	**3.1 ns**	363.7 **	0.139 ***
*VRN-H1*.T	1	0.001 ns	99.4 ***	2.8 ns	0.6 ns	0.043 ns
*VRN-H2*.T	1	0.082 *	11.8 ns	2.9 ns	4.5 ns	0.177 ***
*PPD-H1*.T	1	0.177 **	342.4 ***	1.3 ns	70.5 ns	0.282 ***
*VRN-H1*. *VRN-H2*.T	1	0.006 ns	0.2 ns	0.6 ns	42.4 ns	0.005 ns
*VRN-H1*.*PPD-H1*.T	1	0.068 *	125.3 ***	8.6 ns	2,353.2 ***	0.416 ***
*VRN-H2.PPD-H1*.T	1	0.009 ns	0.1 ns	4.2 ns	70.7 ns	0.028 ns
*VRN-H1*.*VRN-H2*.*PPD-H1*.T	1	0.034 ns	12.9 ns	1.3 ns	3.9 ns	0.024 ns
Genotype*Vernalization	**5**	**0.026 ns**	**6.9 ns**	12.1 ***	**141.2 ns**	**0.023 ns**
*VRN-H1*.V	1	0.017 ns	2.7 ns	6.3 ns	51.1 ns	0.088 *
*VRN-H2*.V	1	0.002 ns	0.2 ns	2.7 ns	0.1 ns	0.021 ns
*PPD-H1*.V	1	0.012 ns	21.7 *	32.3 ***	277.8 ns	0.000 ns
*VRN-H1*.*VRN-H2*.V	0					
*VRN-H1*.*PPD-H1*.V	1	0.050 ns	9.7 ns	17.8 **	180.0 ns	0.007 ns
*VRN-H2*. *PPD-H1*.V	1	0.050 ns	0.4 ns	1.6 ns	196.9 ns	0.001 ns
*VRN-H1*.*VRN-H2*. *PPD-H1*.V	0					
Genotype*Temperature* **Vernalization**	**5**	0.133 ***	31.7 ***	**4.8 ns**	**129.4 ns**	0.070 **
*VRN-H1*.T.V	1	0.442 ***	103.0 ***	14.3 *	195.7 ns	0.176 ***
*VRN-H2*.T.V	1	0.157 **	18.6 *	3.4 ns	239.5 ns	0.048 ns
*PPD-H1*.T.V	1	0.000 ns	17.5 ns	0.0 ns	147.8 ns	0.032 ns
*VRN-H1*.*VRN-H2*.T.V	0					
*VRN-H1*.*PPD-H1*.T.V	1	0.005 ns	3.3 ns	3.5 ns	22.3 ns	0.012 ns
*VRN-H2*. *PPD-H1*.T.V	1	0.062 *	16.1 ns	2.9 ns	41.5 ns	0.081 *
*VRN-H1*.*VRN-H2*. *PPD-H1*.T.V	0					
**Residual 2**	**72**	**0.016**	**4.6**	**2.7**	**132.5**	**0.015**

TGW, thousand grain weight. ns, *, **, ***, indicates non-significant, and significance at the 0.05, 0.01, and 0.001 probability level, respectively. C01 and C02, unvernalized treatments, set as missing values. In bold, values of the main factors of the analyses.

**FIGURE 5:**
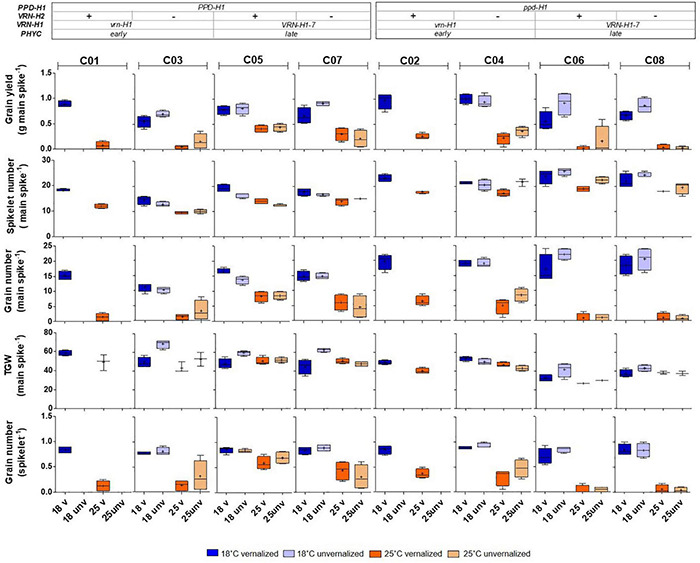
Inflorescence traits corresponding to the main spike: grain yield, spikelet number, grain number, thousand grain weight (TGW), and grain number per spikelet of eight barley NILs subjected to non-vernalized and vernalized conditions and grown at 18 and 25°C. The genetic composition of the lines is described in the top inset.

Some *VRN-H1* and *VRN-H2* effects on yield and components were significant, particularly the main effects on grain yield and spikelets per spike but, as for the developmental traits, the most important effects on these traits were also caused by *PPD-H1*, except for grain yield per main spike (Table 3). Recessive *ppd-H1* lines always had more spikelets per spike than their respective *PPD-H1* counterparts, while the trend for TGW was opposite (higher TGW for dominant *PPD-H1* lines) across all treatments (Figure 5).

In addition, the interactions between *PPD-H1* and *VRN-H1*/*PHYC* and *PPD-H1* with temperature (interactions *VRN-H1* × *PPD-H1, PPD-H1* × T, *VRN-H1* × *PPD-H1* × T), were significant for grain yield or several yield components (Table 3). The effect of interactions between *PPD-H1* and temperature and *PPD-H1* and *VRN-H1* (when testable) were confirmed in partial analyses involving only lines C03-C08, and lines C05-C08, so these effects were independent of growth habit. The most striking result was the large interaction between *VRN-H1* and *PPD-H1* for yield of the main spike (Table 3). Recessive *ppd-H1* lines had higher yield in *vrn-H1*/*PHYC-e* background across all treatments, and dominant *PPD-H1* lines yielded best in *VRN-H1-7*/*PHYC-l* lines overall. However, this last observation was caused by the clear yield advantage of dominant *PPD-H1* lines at 25°C, whereas at 18°C there was no advantage of either allele. All lines showed a striking yield reduction at 25°C, but the largest reductions occurred in haplotypes *vrn-H1/PPD-H1* and *VRN-H1-7/ppd-H1*. They were caused by poorer seed set and, to a lesser extent, by a reduced TGW only in *VRN-H1-7/ppd-H1* lines (Figure 5).

The phenological traits of these isolines at 18°C are good predictors of their development at 25°C (correlation coefficients of 0.96 and 0.99 for Z49 in unvernalized and vernalized plants, respectively), but the predictability of yield at high temperature, based on the 18°C performance was poor (correlations of 0.44 for vernalized plants, and 0.08 for unvernalized ones). For yield components, the number of spikelets per main spike and TGW present the largest correlation coefficients between temperatures (0.93 and 0.68, respectively), whereas the correlations for grain number per spike and grain yield per main spike were close to 0. The duration of the phase until the appearance of the first node (Z31), determines the number of spikelets. The lines with the insensitive *ppd-H1* allele presented a delayed appearance of the first node and, fittingly, they are characterized by a higher spikelet number per spike than lines with the sensitive *PPD-H1* alleles. However, insensitive *ppd-H1* lines also presented lower grain number in the background of spring *VRN-H1-7* alleles at high temperature, indicating a reduced spike fertility.

### Gene Expression

**FIGURE 6:**
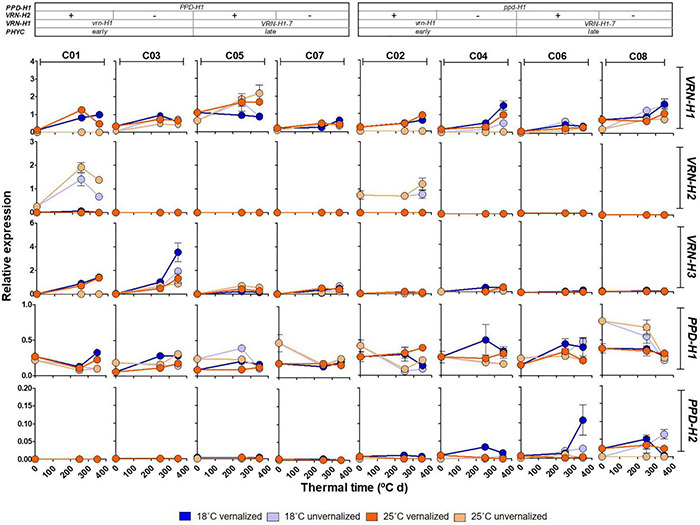
Expression patterns of *VRN-H1*, *VRN-H2*, *VRN-H3*, *PPD-H1* and *PPD-H2* at three different times (0, 250 and 350°C d), for the eight barley NILs (C01-C08) subjected to non-vernalized and vernalized conditions and grown at 18 and 25°C. The genetic composition of the lines is described in the top inset.

As expected, winter barleys (C01 and C02) exhibited *VRN-H2* expression mostly in unvernalized plants, in absence of its *VRN-H1* repressor (Figure 6). Initially, *VRN-H2* transcripts were not detected in spring barleys in this experiment. However, we had previous data indicating that this gene was expressed in spring lines C05 and C06 (Monteagudo, personal communication). We grew these lines under two temperatures and in absence of vernalization in a separate experiment. We found a small but clear *VRN-H2* expression, apparently following a circadian rhythm, with maximum expression at the end of the day period, higher in C05 than in C06 (Supplementary Figure 8). Therefore, a phenotypic effect of *VRN-H2* expression in these spring lines cannot be ruled out.

All the lines had the same *VRN-H3* and *PPD-H2* alleles. However, their expression was strongly dependent on the *PPD-H1* allele present, with lines carrying the sensitive allele showing higher *VRN-H3* expression. Expression of *PPD-H2*, the “short-day sensitivity” gene was detected, although the experiment was carried under long days. Contrary to *VRN-H3*, *PPD-H2* expression was higher in recessive *ppd-H1* lines (Figure 6). *PPD-H2* expression also varied widely with the experimental factors, with highest expression in vernalized plants at 18°C.

Patterns of *PPD-H1* expression were highly variable across genotypes (Figure 6). Overall, the insensitive allele of *PPD-H1* showed higher expression than the sensitive one (Figure 6 and Supplementary Table 6). These results may be affected by the specific sampling time, as the circadian rhythm of the two *PPD-H1* alleles may reach expression peaks at different times (Campoli et al., 2012), as also hinted in our result with sequential samples at 18°C (Supplementary Figure 8). *PPD-H1* expression is modulated by the allele present at *VRN-H1*/*PHYC*, as indicated by a significant interaction (Supplementary Table 6).

Gene expression of flowering promoters *VRN-H1* and *VRN-H3* was negatively correlated with development variables (Supplementary Figure 5), whereas the opposite was true for that of *PPD-H1* and *PPD-H2* (which showed high interdependence, as mentioned before). Yield and grain number, on the other hand were rather independent of development and gene expression variables. Late lines showed an increased number of spikelets per spike, but lower TGW (Figure 5).

## Discussion

This set of isogenic lines constitutes an excellent genetic screen to test the joint effects of developmental genes. The components of the vernalization and photoperiod pathways in barley are rich in interactions and, therefore, studying their effects in isolation may not reveal their roles in full. In fact, this study has unraveled a complex grid of interactions between the main genes of the vernalization and photoperiod pathways, and the environmental conditions, some of them unexpected.

### Which Gene Has the Largest Effect on Development?

The performance of winter lines C01 and C02, was in agreement with the epistatic mode of action of the vernalization mechanism in winter varieties proposed by Trevaskis et al. (2006). Aside from the failure of winter lines to progress toward flowering without vernalization, *PPD-H1* had the largest effect on development, irrespective of temperature. This finding is in accordance with other studies carried out with barley (Turner et al., 2005; Digel et al., 2016; Wiegmann et al., 2019; Gol et al., 2021).

We could not separate the effects of *VRN-H1* and *PHYC* fully. We could deduce, however, that the strong earliness effect induced by *PHYC-e* (Nishida et al., 2013; Pankin et al., 2014) was not able to override the need for vernalization of winter lines C01 and C02, as they did not progress toward reproductive development when not vernalized. The protein-protein interaction between PHYC and VRN2 described in wheat by Shaw et al. (2020) may have played a role, as *VRN-H2* was expressed in abundance only in those genotypes and condition.

We have observed a larger role of vernalization on the initial phase of the cycle, until onset of stem elongation (Z31), than on the late reproductive phase (Z31-49). Yet, vernalization effects were still appreciable in the second phase, as previously reported by González et al. (2002). In contrast, temperature affected the duration of the entire cycle, but more markedly the late reproductive phase, in accordance with previous findings in wheat (Slafer and Rawson, 1994). However, Karsai et al. (2013) observed temperature effects on barley development at all growth stages, particularly around the appearance of the first node.

Apparently, vernalization acted as a stabilizing factor for the development of the lines, having more or less effect on development depending on the genetic background, and counteracted the effect of high temperature on both phenology and yield. Vernalized lines had a more stable behavior across temperatures, whereas unvernalized plants suffered developmental delays at high temperature during the two stages considered. In every case, delays in development were related with longer phyllochrons. This observation may be relevant to barley grown in autumn sowings. Cold periods are long enough to provide at least some vernalization in most areas where it is practiced.

Our high temperature treatment, 25°C is likely above the optimum temperature for barley growth. The effect of this treatment varied among genotypes and growth phases, which may be related to the reported differences in optimum temperatures for these phases in wheat and barley (Jacott and Boden, 2020). Optimum temperatures up to Z31 are lower than after that stage, and the larger effect at this stage observed for some lines could be related to a wider distance between the optimum temperature and our high temperature treatment. We found that high temperatures delayed flowering time in unvernalized plants, except line C03. It has been reported that high temperatures delay development of barley genotypes carrying the recessive *ppd-H1* allele (Ejaz and von Korff, 2017), which agrees with our own results. However, when vernalized, the delay in development of *ppd-H1* plants occurred only in spring barleys (C06 and C08, compared to C05 and C07, respectively). Other stress conditions, like drought, also affect spring barleys carrying insensitive *ppd-H1* alleles, by delaying flowering time (Gol et al., 2021). It is sensible to speculate whether this allele presents intrinsic disadvantages under abiotic stress conditions.

We have demonstrated that the previously reported effect of temperature on *PPD-H1* alleles is not constant. It depends, to a large extent, on interactions between environmental conditions (presence/absence of vernalization and ambient temperature), and the haplotype at the vernalization genes.

Traditionally, spring cultivars do not present any of the winter alleles at *VRN-H1* and *VRN-H2* (in a few cases, they carry just one of the two). These results indicate that a judicious use of allelic diversity at the vernalization genes, in combination with the allele at *PPD-H1*, would provide ample options to modulate plant development, and fine tuning it according to the expected temperatures and sowing times. Different haplotypes will either induce a longer vegetative phase, or a longer phase after onset of stem elongation. Which strategy is best to enhance grain yield production at each environment, will have to be ascertained with further research involving modeling for future conditions. Then, plant breeders will make use of this toolbox of alleles and known interactions with environment to construct ideotype cultivars specific to each agroecological region.

### The Presence of an Active *VRN-H2* Affects the Development of Spring Lines

One of the novel results of this work was the detection of an effect of *VRN-H2* on development, partly through the interaction *VRN-H2* × *PPD-H1*, which was particularly striking in the case of spring lines (C05-C08). According to the current consensus, the spring *VRN-H1* allele they carry should be constitutively expressed, without the need of external cues, and should repress the expression of *VRN-H2*. Initially, we did not detect clear signs of expression of *VRN-H2* in these lines (Figure 6). However, those samples represent a single time of the day, and we could have missed the moment of peak expression. We did a new experiment growing unvernalized lines C05 and C06, at 16 h and 18°C or 25°C, and checked gene expression (for *VRN-H2* and other genes) at several time points of the day in 13-day old plants. The results, presented in Supplementary Figure 8, indicated that there was feeble but unequivocal *VRN-H2* expression in both lines, more at 18°C than at 25°C. This demonstrates that *VRN-H2* is expressed in spring lines under long days, and could be responsible for the effects observed. The relationship of the *VRN-H2* by *PPD-H1* interaction with the duration of growth until Z31 could be related with the existence of regulatory feedback loops between *VRN-H2*, *PPD-H1* and *HvCO2*, described by Mulki and von Korff (2016).

The development of line C06, *ppd-H1*/*VRN-H2* is particularly interesting, as it represents an alternative format to the typical pattern of spring cultivars (line C08). C06 ideotype may have an agronomic merit. It is earlier than C08, but has a similar duration of the phase after the onset of stem elongation as C08, which is crucial for the determination of the potential number of grains in the spike. Its earliness is due to a shorter phase until Z31. This could be a way of shortening the cycle of spring cultivars without compromising yield potential, although its yielding ability is heavily impaired at high temperature.

### Growth Cycle of Spring (*ppd-H1* Recessive) Lines Is Modified by Vernalization Through Changes in Phyllochron

Vernalization, optimal temperature, and the presence of an ancestral *PPD-H1* allele, are all stabilizing factors for barley development. Alterations in any of them cause a wide variety of responses.

One of the unexpected results of this study was finding a *PPD-H1* × V interaction for the duration of period Z31-49, in all analyses of variance performed. It is remarkable that it was one of the most relevant sources of variance in the ANOVA restricted to the spring lines (C05-C08). The spring lines with the recessive *ppd-H1* allele were significantly earlier in reaching flowering when not subjected to vernalization than when vernalized (89°C d). The difference was entirely due to a longer late reproductive period of *ppd-H1* recessive lines when vernalized, related to a longer phyllochron (16% longer than without vernalization). We cannot discard that there are other genes acting in the vernalization pathway still present in the genome of these isolines, interacting with vernalization and *PPD-H1*, to produce the results observed.

### *PPD-H1* and *VRN-H1* Interaction Affects Grain Yield

Estimation of grain yield and its components in plants grown in growth chambers cannot be extrapolated to field conditions. Nonetheless, we expect that the trends observed could be indicative of what can also be expected in the field. We confirmed a drastic reduction of grain yield, spikelet number and grain number, in the eight lines, as a consequence of exposure to a constant high temperature throughout the cycle. In fact, short or prolonged periods of high temperatures are known to reduce barley yield (Hemming et al., 2012; Jacott and Boden, 2020), which has been confirmed in field experiments applying heat stress, both on wheat (Wollenweber et al., 2003; García et al., 2015; Elía et al., 2018), and barley (García et al., 2016), considerably reduced grain number and grain weight.

Lines carrying the sensitive *PPD-H1* allele presented lower spikelet number than lines with insensitive *ppd-H1* alleles, irrespective of temperature and vernalization treatments, thus showing a lower yield potential. At 18°C, plants with *ppd*-H1 alleles generated more grains per spike and yielded more. At 25°C, however, spike fertility and grain filling of *ppd-H1* plants collapsed. The number of grains produced per spike dropped dramatically, and the few grains produced were lighter, compared to *PPD-H1* carrying plants. Ejaz and von Korff (2017), also found that high ambient temperatures caused a larger reduction of seeds per spike in lines carrying insensitive *ppd-H1* alleles, in comparison with those carrying sensitive *PPD-H1* alleles. However, in our case, the effect was only evident in lines carrying also the spring *VRN-H1-7* allele.

The consequences of these phenomena cannot be extrapolated directly to field-grown barley. However, there is sound evidence on the large impact of *PPD-H1* alleles on grain yield depending on environmental conditions. Wiegmann et al. (2019) found that, where earliness was beneficial, the sensitive *PPD-H1* allele provided a yield advantage. Gol et al. (2021) reported a larger yield stability under drought for plants with the sensitive allele. The study of yield impacts of this gene and its orthologs in relation with temperature should be a priority in cereal research.

### *PPD-H1* Alleles Associated to Transcript Levels of *VRN-H3* and *PPD-H2*

The interactions between *VRN-H*2 and *PPD-H1*, and of these two genes with *VRN-H1* had a large influence on *VRN-H1* expression. The highest *VRN-H1* expression in line C05 seemed to underpin these interactions (Supplementary Table 6). There is no clear explanation for this strikingly high level of *VRN-H1* expression in this line, across all four treatments. Oliver et al. (2013) reported a higher expression potential for *VRN-H1-7*, compared to other alleles but, in our results, this was only true for line C05 and not for the other three lines with this allele. Therefore, we hypothesize that the higher expression of this *VRN-H1* allele is not constitutive, and that it depends on the genetic background. Usually, this higher expression should result in upregulation of *VRN-H3* (Deng et al., 2015), but this is not clearly observed in C05. This line carries, besides a spring allele at *VRN-H1*, a winter *VRN-H2* allele and a sensitive *PPD-H1* allele. According to Mulki and von Korff (2016), a sensitive *PPD-H1* allele up-regulates *VRN-H2* expression. Additionally, we have detected expression of *VRN-H2* in C05 (Supplementary Figure 8). Therefore, we could speculate on the presence of a feedback loop mechanism inducing more *VRN-H1* expression in C05 to down-regulate the *VRN-H2* present in this line.

*PPD-H2* was expressed in some lines, although at low levels, despite the long photoperiod. However, it is controlled by day length only indirectly, with other genes acting as intermediary (Casao et al., 2011). Actually, observing its expression in long days, as in our experiment, is not new (Kikuchi et al., 2009; Casao et al., 2011; Monteagudo et al., 2019, 2020). The clear dependence of its expression on the *PPD-H1* allele observed here, was already pointed out by Mulki et al. (2018).

As expected, lines with sensitive *PPD-H1* alleles showed higher transcript levels of *VRN-H3* and flowered earlier compared with recessive *ppd-H1* alleles (Turner et al., 2005).

The main genetic feature of spring cultivars from Central Europe is the presence of the insensitive *ppd-H1* allele (Jones et al., 2011; Russell et al., 2016; Bustos-Korts et al., 2019). The appearance of the insensitive *ppd-H1* allele allowed the expansion of the crop to areas in which late winter or spring sowings were required to escape the harsh winter conditions (Jones et al., 2008; Wiegmann et al., 2019). Most spring cultivars, however, also carry the active *PPD-H2* allele (early), and haplotype III at gene *HvCEN* (late). This combination of alleles may have an adaptive purpose, which has not been explained yet. The presence of the active *PPD-H2* allele, the “short photoperiod sensitivity” gene seemed irrelevant in spring sowings, because these crops complete their cycles under long days. The fact that *PPD-H2*, a known promoter of spikelet initiation (Mulki et al., 2018) is expressed in long days, and at higher levels in the presence of the insensitive *ppd-H1* allele, suggests an active role of *PPD-H2* in promotion of development even in spring sowings.

In conclusion, our study provides useful tools to better understand the physiological and molecular responses of barley at high ambient temperatures and contrasting vernalizing treatments, through several allelic combinations of *VRN-H1*, *VRN-H2* and *PPD-H1* genes. New contributions shown here might be important for breeders to develop barley varieties with improved resilience under future climate conditions.

## Data Availability Statement

Publicly available datasets were analyzed in this study. This data can be found here: DIGITAL.CSIC, at http://hdl.handle.net/10261/256755. Further inquiries can be directed to the corresponding author/s.

## Author Contributions

IK, AC, and EI: conceptualization, methodology, and writing—review and editing. TK, IK, and HO: investigation. HO, AC, and EI: formal analysis. AC and EI: supervision and funding acquisition. HO: visualization. HO and EI: writing—original draft preparation. All authors read and approved the final manuscript.

## Conflict of Interest

The authors declare that the research was conducted in the absence of any commercial or financial relationships that could be construed as a potential conflict of interest.

## Publisher’s Note

All claims expressed in this article are solely those of the authors and do not necessarily represent those of their affiliated organizations, or those of the publisher, the editors and the reviewers. Any product that may be evaluated in this article, or claim that may be made by its manufacturer, is not guaranteed or endorsed by the publisher.
