# Supplementary material for: Responses of Barley to High Ambient Temperature Are Modulated by Vernalization
Source: Front Plant Sci. 2022 Jan 25;12:776982. doi: 10.3389/fpls.2021.776982 (PMC8822234; doi:10.3389/fpls.2021.776982)
Supplement: Supplementary file 1 [file Data_Sheet_1.docx]

Supplementary Material

# Supplementary Figures and Tables

## Supplementary Figures

**Supplementary Figure 1.** Chromosomal locations of introgressed segments in near isogenic lines C01 **(A)**, C02 **(B)**, C03 **(C)**, C04 **(D)**, C05 **(E)**, C06 **(F)**, C07 **(G)** and C08 **(H)**. Lines were genotyped with the Barley 50k iSelect SNP Array (Bayer et al., 2017). Target regions highlighted in black; other markers different from the recurrent line (C03) highlighted in red.

**Supplementary Figure 2. (A)** Days to flowering of the near isogenic lines C01-C08. Flowering time was recorded for at least 10 plants per genotype. Previously, plants were vernalized during 8 weeks (C01 and C02) or 7 weeks (C03-C08) at 4°C. **(B)** Images of NILs carrying *PPD-H1* alleles (C01, C03, C05 and C07) and NILs with *ppd-H1* alleles (C02, C04, C06 and C08). Photos were taken 36 days (C01 and C02) or 41 days (C03-C08) after transplant to glasshouse. At that moment, plants were subjected to daylength of 11h.

**Supplementary Figure 3.** Proportion of sums of squares explained by each factor in an analysis of variance of days to flowering time (Z49), duration of the phase from the onset of experiment to appearance of first node (Z31), duration of the phase from onset of stem elongation to flowering time (Z31-49), final leaf number and phyllochron, considering the eight NILs.

**Supplementary Figure 4.** Apex development in winter NILs (*vrn-H1, VRN-H2)* grown under the four experimental conditions, combination of 18 or 25°C, and presence/absence of vernalization. Photos were taken on the 27^th^ day after the beginning of temperature treatments.

**Supplementary Figure 5.** Correlation network and multiple factorial analysis (MFA) of all variables considered in the experiment. (A) Correlation network based on pairwise correlations (*P*<0.05). The colours grey, white and black of the nodes represent phenology-development, yield and yield components and gene expression variables, respectively. Line thickness reflects the magnitude of the correlation coefficients (brown = positive; blue = negative). (B) Graphical representation of Multiple Factorial Analysis (MFA) coloured according to variable groups. Z49: appearance of the awns just visible above the last leaf sheath (flowering time), Z31: appearance of the first node at the base of the main stem, Z31-49: late reproductive phase, FLN: final leaf number, Phyll: phyllochron, GY: grain yield per main spike, GN: grain number per main spike, SPIK: spikelet number, GN_SPIK: grain number per spikelet, TGW: thousand grain weight, e*VRN-H1*: expression of *VRN-H1*, e*VRN-H2*: expression of *VRN-H2*, e*VRN-H3*: expression of *VRN-H3*, e*PPD-H1*: expression of *PPD-H1* and e*PPD-H2*: expression of *PPD-H2*.

**Supplementary Figure 6.** Differences in thermal time between NILs carrying insensitive *ppd-H1* and sensitive *PPD-H1* alleles, on the three developmental phases, flowering time (Z49), onset of stem elongation (Z31) and late reproductive phase (Z31-49).

**Supplementary Figure 7.** Relationship between cumulative leaf appearance in the main shoot (Haun stage) and thermal time from onset of temperature treatments for the eight NILs.

**
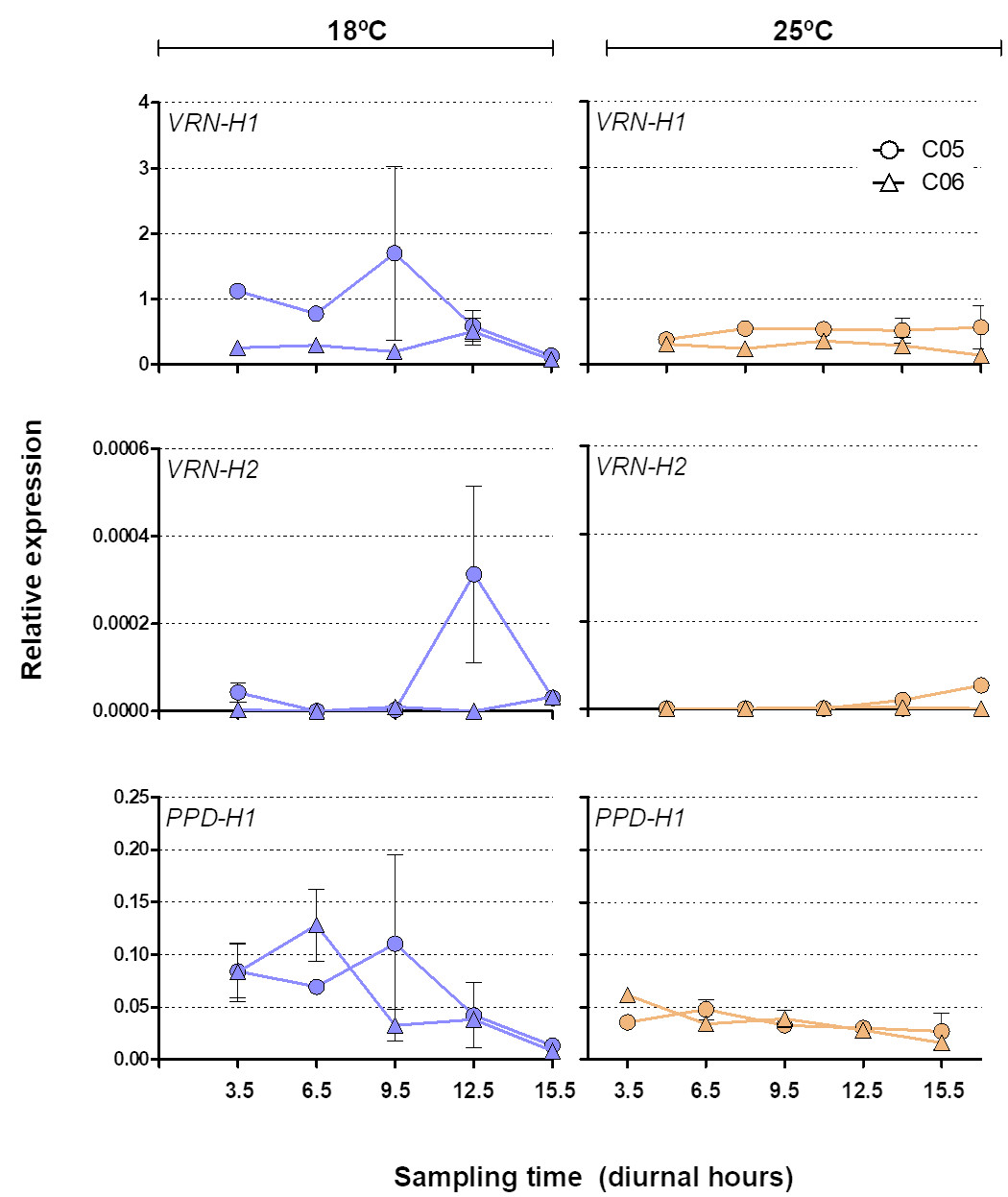
**

**Supplementary Figure 8.** Gene expression patterns of *VRN-H1*, *VRN-H2*, and *PPD-H1* at five different times of the day (3.5, 6.5, 9.5, 12.5, 15.5 h after lights were turned on), for NILs C05 and C06 grown at 18 and 25°C, without vernalization. Plants were kept under 16 h day/8 h night for 13 days until sampling. Mean values and standard errors of the means of relative expression of three biological replicates.

## Supplementary Tables

**Supplementary Table 1.** Mean squares of the analyses of variance with 8 NILs, including estimated values of Z49 and Z31 for non-vernalized C01 and C02.

| Source of variation | df | Z49  (ºC d) | Z31  (°C d) | FLN  (leaves) | Phyllochron  (°C d leaf-^1^) |
| --- | --- | --- | --- | --- | --- |
| **Temperature (T)** | **1** | **764350 ***** | **378151 ***** | **0.94 *** | **5774 ***** |
| **Vernalization (V)** | **1** | **5087877 ***** | **7741898 ***** | **53.82 ***** | **2241 ***** |
| **Temperature.Vernalization** | **1** | **609857 ***** | **472301 ***** | **5.69 ***** | **1016 ***** |
| **Residual 1** | **12** | **818** | **111** | **0.17** | **15** |
| **Genotype** | **7** | **2353044 ***** | **3333860 ***** | **33.56 ***** | **4182 ***** |
| *VRN-H1* | 1 | 1973249 *** | 6988488 *** | 18.75 *** | 512 *** |
| *VRN-H2* | 1 | 5046015 *** | 7466389 *** | 48.75 *** | 2669 *** |
| *PPD-H1* | 1 | 2761070 *** | 437742 *** | 73.50 *** | 16169 *** |
| *VRN-H1*.*VRN-H2* | 1 | 6153661 *** | 8308638 *** | 76.57 *** | 3550 *** |
| *VRN-H1*.*PPD-H1* | 1 | 387700 *** | 29178 *** | 7.50 *** | 5490 *** |
| *VRN-H2*.*PPD-H1* | 1 | 112461 *** | 101142 *** | 1.32 *** | 840 *** |
| *VRN-H1*.*VRN-H2*.*PPD-H1* | 1 | 37154 *** | 5444 *** | 8.50 *** | 42 * |
| **Genotype*Temperature** | **7** | **78492 ***** | **89192 ***** | **0.18 ns** | **365 ***** |
| *VRN-H1*.T | 1 | 92110 *** | 220919 *** | 0.01 ns | 57 ** |
| *VRN-H2*.T | 1 | 127183 *** | 198038 *** | 0.07 ns | 121 *** |
| *PPD-H1*.T | 1 | 98767 *** | 33462 *** | 0.01 ns | 1646 *** |
| *VRN-H1*.*VRN-H2*.T | 1 | 195649 *** | 141666 *** | 0.07 ns | 227 *** |
| *VRN-H1*.*PPD-H1*.T | 1 | 12443 *** | 43 ns | 0.94 *** | 429 *** |
| *VRN-H2*.*PPD-H1*.T | 1 | 3735 ** | 1173 ** | 0.07 ns | 72 ** |
| *VRN-H1*.*VRN-H2*.  *PPD-H1*.T | 1 | 19555 *** | 29044 *** | 0.07 ns | 2 ns |
| **Genotype*Vernalization** | **7** | **2286499 ***** | **3147773** *** | **20.45 ***** | **1990 ***** |
| *VRN-H1*.V | 1 | 6362028 *** | 8338832 *** | 53.82 *** | 9237 *** |
| *VRN-H2*.V | 1 | 4487395 *** | 6721778 *** | 46.32 *** | 1324 *** |
| *PPD-H1*.V | 1 | 20015 *** | 30362 *** | 0.01 ns | 588 *** |
| *VRN-H1*.*VRN-H2*.V | 1 | 5017096 *** | 6884571 *** | 41.63 *** | 1576 *** |
| *VRN-H1*.*PPD-H1*.V | 1 | 8836 *** | 3377 *** | 0.01 ns | 287 *** |
| *VRN-H2*.*PPD-H1*.V | 1 | 37884 *** | 22052 *** | 0.94 *** | 155 *** |
| *VRN-H1*.*VRN-H2*.*PPD-H1*.V | 1 | 72236 *** | 33436 *** | 0.38 * | 766 *** |
| **Genotype*Temperature***  **Vernalization** | **7** | **76574 ***** | **96369 ***** | **0.96 ***** | **105 ***** |
| *VRN-H1*.T.V | 1 | 194506 *** | 199686 *** | 0.01 ns | 293 *** |
| *VRN-H2*.T.V | 1 | 117781 *** | 145582 *** | 0.19 ns | 34 * |
| *PPD-H1*.T.V | 1 | 10433 *** | 27718 *** | 5.69 *** | 1 ns |
| *VRN-H1*.*VRN-H2*.T.V | 1 | 174664 *** | 289210 *** | 0.19 ns | 1 ns |
| *VRN-H1*.*PPD-H1*.T.V | 1 | 11819 *** | 1035 ** | 0.63 *** | 192 *** |
| *VRN-H2*.*PPD-H1*.T.V | 1 | 3649 ** | 11352 *** | 0.01 ns | 62 ** |
| *VRN-H1*.*VRN-H2*.  *PPD-H1*.T.V | 1 | 23169 *** | 0 ns | 0.01 ns | 153 *** |
| **Residual 2** | **84** | **545** | **146** | **0.07** | **7** |

Z49: days to awn appearance (flowering time), Z31: days to first node appearance; FLN: final leaf number.

ns, *,**,***, indicates non-significant and significance at the 0.05, 0.01, and 0.001 probability level, respectively.

**Supplementary Table 2.** Mean squares of the analyses of variance restricted to NILs C03 to C08. Inflorescence traits measured at the main spike of each plant.

| Source of variation | df | Z49  (°C d) | Z31  (°C d) | Z31-49  (°C d) | FLN  (#leaves) | Phyll  (°C d) | Grain yield.  (g) | Grain # | Spikelet # | TGW  (g) | Grain #  spikelet^-1^ |
| --- | --- | --- | --- | --- | --- | --- | --- | --- | --- | --- | --- |
| **Temperature (T)** | **1** | **192627 ***** | **25521***** | **77919 ***** | **1.0 ***** | **4464 ***** | **8.21 ***** | **3675 ***** | **349 ***** | **4692 ***** | **7.28 ***** |
| **Vernalization (V)** | **1** | **1076 ns** | **4924 ***** | **10605 **** | **0.2 ns** | **79 **** | **0.25 **** | **9 ns** | **4 ns** | **808 *** | **0.04 ns** |
| **T.V** | **1** | **108424 ***** | **40313 ***** | **16511 ***** | **3.4 ***** | **604 ***** | **0.06 ns** | **0 ns** | **13 ns** | **433 ns** | **0.00 ns** |
| **Residual 1** | **12** | **972** | **188** | **946** | **0.0** | **8** | **0.02** | **6** | **3** | **136** | **0.02** |
| **Genotype (G)** | **5** | **774683 ***** | **120042 ***** | **320205 ***** | **20.0** | **5283 ***** | **0.21 ***** | **74 ***** | **274 ***** | **2385 ***** | **0.27 ***** |
| *VRN-H1* | 1 | 612829 *** | 17908 *** | 421219 *** | 8.3 *** | 4081 *** | 0.00 ns | 15 ns | 213 *** | 1566 *** | 0.01 ns |
| *VRN-H2* | 1 | 27454 *** | 11250 *** | 3555 * | 1.6 *** | 31 ** | 0.04 ns | 12 ns | 9 ns | 83 ns | 0.04 ns |
| *PPD-H1* | 1 | 3137237 *** | 540808 *** | 1072940 *** | 88.2 *** | 19043 *** | 0.00 ns | 67 *** | 1114 *** | 6590 *** | 0.35 *** |
| *VRN-H1*. *PPD-H1* | 1 | 85730 *** | 420 ns | 98147 *** | 0.3 ** | 2634 *** | 0.98 *** | 266 *** | 15 * | 3517 *** | 0.81 *** |
| *VRN-H2*. *PPD-H1* | 1 | 10167 *** | 29828 *** | 5166 ** | 1.6 *** | 630 *** | 0.02 ns | 12 ns | 20 ** | 167 ns | 0.11 ** |
| **G*T** | **5** | **28863 ***** | **23586 ***** | **11539 ***** | **0.1 ns** | **505 ***** | **0.07** | **107** | **3 ns** | **446 ***** | **0.17 ***** |
| *VRN-H1*.T | 1 | 6141 ** | 2346 *** | 16078 *** | 0.0 ns | 61 *** | 0.00 ns | 48 ** | 3 ns | 8 ns | 0.00 ns |
| *VRN-H2*.T | 1 | 3672 * | 2355.0 *** | 11908 *** | 0.1 ns | 340 *** | 0.07 ns | 8 ns | 3 ns | 10 ns | 0.12 ** |
| *PPD-H1*.T | 1 | 131320 *** | 48133 *** | 20446 *** | 0.0 ns | 1932 *** | 0.27 *** | 408 *** | 0 ns | 377 ns | 0.41 *** |
| *VRN-H1*.*PPD-H1*.T | 1 | 82 ns | 6117 *** | 7613 ** | 0.3 ** | 166 *** | 0.00 ns | 61 *** | 4 ns | 1783 *** | 0.28 *** |
| *VRN-H2*.*PPD-H1*.T | 1 | 3099 * | 9272 *** | 1650 ns | 0.0 ns | 26 * | 0.00 ns | 8 ns | 5 ns | 54 ns | 0.05 ns |
| **G*V** | **5** | **31925 ***** | **27866 ***** | **14971 ***** | **0.3 **** | **698 ***** | **0.03 ns** | **9 ns** | **13 ***** | **134 ns** | **0.02 ns** |
| *VRN-H1*.V | 1 | 78519 *** | 52066 *** | 2708 ns | 0.3 ** | 2248 *** | 0.01 ns | 3 ns | 3 ns | 10 ns | 0.05 ns |
| *VRN-H2*.V | 1 | 3672 ** | 487 ns | 4080 * | 0.0 ns | 6 ns | 0.01 ns | 2 ns | 2 ns | 25 ns | 0.03 ns |
| *PPD-H1*.V | 1 | 252 ns | 52799 *** | 60351 *** | 0.2 ns | 205 *** | 0.01 ns | 30 ** | 44 *** | 249 ns | 0.00 ns |
| *VRN-H1*.*PPD-H1*.V | 1 | 70720 *** | 33388 *** | 6924 ** | 1.0 *** | 913 *** | 0.06 ns | 3 ns | 8 ns | 336 ns | 0.00 ns |
| *VRN-H2*.*PPD-H1*.V | 1 | 2748 * | 590 ns | 791 ns | 0.1 ns | 116 *** | 0.05 ns | 5 ns | 9 ns | 50 ns | 0.00 ns |
| **G*T*V** | **5** | **10860 ***** | **10444 ***** | **11224 ***** | **1.1 ***** | **137 ***** | **0.04 ns** | **12 *** | **2 ns** | **58 ns** | **0.02 ns** |
| *VRN-H1*.T.V | 1 | 2438 ns | 109 ns | 3579 * | 0.2 ns | 279 *** | 0.10 * | 29 ** | 4 ns | 11 ns | 0.05 ns |
| *VRN-H2*.T.V | 1 | 2793 * | 12204 *** | 3320 * | 0.0 ns | 13 ns | 0.03 ns | 1 ns | 1 ns | 60 ns | 0.01 ns |
| *PPD-H1*.T.V | 1 | 14031 *** | 33885 *** | 4307 * | 5.0 *** | 2 ns | 0.00 ns | 11 ns | 1 ns | 215 ns | 0.00 ns |
| *VRN-H1.PPD-H1*.T.V | 1 | 30824 *** | 289 ns | 25140 *** | 0.5 ** | 384 *** | 0.02 ns | 8 ns | 1 ns | 0 ns | 0.00 ns |
| *VRN-H2.PPD-H1*.T.V | 1 | 4214 * | 5732 *** | 19775 *** | 0.0 ns | 10 ns | 0.03 ns | 12 ns | 3 ns | 5 ns | 0.05 ns |
| **Residual 2** | **60** | **613** | **184** | **741** | **0.0** | **5** | **0.02** | **5** | **3** | **122** | **0.02** |

Z49: days to awn appearance (flowering time), Z31: days to first node appearance; FLN: final leaf number; Phyll: phyllochron; Grain #: grain number per main spike; Spikelet #: spikelet number per main spike; TGW: thousand grain weight; Grain #.spikelet^-1^: grain number per spikelet.

| Source of variation | df | Z49  (°C d) | Z31  (°C d) | Z31-49  (°C d) | FLN  (#leaves) | Phyll  (°C d) | Grain yield.  (g) | Grain # | Spikelet # | TGW  (g) | Grain #  spikelet^-1^ |
| --- | --- | --- | --- | --- | --- | --- | --- | --- | --- | --- | --- |
| **Temperature (T)** | **1** | **162892 ***** | **10501 ***** | **90676 ***** | **0.6 *** | **3489 ***** | **5.30 ***** | **2862 ***** | **264 ***** | **2943 ***** | **4.91 ***** |
| **Vernalization (V)** | **1** | **35558 ***** | **5542 ***** | **13024 **** | **0.0 ns** | **1189 ***** | **0.21 **** | **2 ns** | **6 ns** | **628 *** | **0.00 ns** |
| **T.V** | **1** | **57768 ***** | **28891 ***** | **4952 *** | **3.1 **** | **109 **** | **0.15 **** | **8 ns** | **3 ns** | **356 ns** | **0.00 ns** |
| **Residual 1** | **12** | **998** | **139** | **891** | **0.1** | **7** | **0.01** | **6** | **3** | **113** | **0.01** |
| **Genotype (G)** | **3** | **882213 ***** | **129184 ***** | **354227 ***** | **22.4 ***** | **6971 ***** | **0.15 ***** | **11 ns** | **218 ***** | **3451 ***** | **0.38 ***** |
| *VRN-H2* | 1 | 27454 *** | 11250 *** | 3555 | 1.6 *** | 31 * | 0.04 ns | 12 ns | 9 * | 83 ns | 0.03 ns |
| *PPD-H1* | 1 | 2609019 *** | 346474 *** | 1053959 *** | 64.0 *** | 20251 *** | 0.40 *** | 8 ns | 625 *** | 10104 *** | 1.01 *** |
| *VRN-H2*. *PPD-H1* | 1 | 10167 *** | 29828 *** | 5166 * | 1.6 *** | 630 *** | 0.02 ns | 12 ns | 20 ** | 167 ns | 0.11 ** |
| **G*T** | **3** | **32478** | **9859** | **13830 ***** | **0.2 ns** | **748 ***** | **0.10 **** | **152 ***** | **3 ns** | **561 ***** | **0.27 ***** |
| *VRN-H2*.T | 1 | 3672 * | 2355 ** | 11908 *** | 0.0 ns | 340 *** | 0.07 ns | 8 ns | 3 ns | 10 ns | 0.12 ** |
| *PPD-H1*.T | 1 | 90662 *** | 17950 *** | 27930 *** | 0.6 ** | 1878 *** | 0.23 ** | 441 *** | 2 ns | 1619 *** | 0.69 *** |
| *VRN-H2*.*PPD-H1*.T | 1 | 3099 * | 9272 *** | 1650 ns | 0.0 ns | 26 ns | 0.00 ns | 8 ns | 5 ns | 54 ns | 0.00 ns |
| **G*V** | **3** | **12619 ***** | **2607 ***** | **22228 ***** | **0.0 ns** | **323 ***** | **0.03 ns** | **12 ns** | **20 ns** | **27 ns** | **0.01 ns** |
| *VRN-H2*.V | 1 | 7386 ** | 487 ns | 4080 * | 0.1 ns | 6 ns | 0.01 ns | 2 ns | 3 ns | 25 ns | 0.02 ns |
| *PPD-H1*.V | 1 | 27723 *** | 6744 *** | 61814 *** | 0.0 ns | 849 *** | 0.05 ns | 30 * | 49 *** | 5 ns | 0.00 ns |
| *VRN-H2*.*PPD-H1*.V | 1 | 2748 * | 590 ns | 791 ns | 0.1 ns | 116 *** | 0.04 ns | 5 ns | 9 * | 50 ns | 0.00 ns |
| **G*T*V** | **3** | **2343 *** | **12556 ***** | **14719 ***** | **1.7 ***** | **43 ***** | **0.02 ns** | **10 ns** | **2 ns** | **69 ns** | **0.02 ns** |
| *VRN-H2*.T.V | 1 | 2793 * | 12203 *** | 3321 ns | 0.0 ns | 13 ns | 0.02 ns | 1 ns | 1 ns | 60 ns | 0.00 ns |
| *PPD-H1*.T.V | 1 | 22 ns | 19733 *** | 21061 *** | 5.1 *** | 107 *** | 0.00 ns | 18 ns | 2 ns | 143 ns | 0.00 ns |
| *VRN-H2*. *PPD-H1*.V.T | 1 | 4214 * | 5731 *** | 19775 *** | 0.0 ns | 10 ns | 0.03 ns | 12 ns | 3 ns | 5 ns | 0.05 ns |
| **Residual 2** | **6** | **667** | **246** | **914** | **0.1** | **8** | **0.02** | **5** | **2** | **102** | **0.01** |

**Supplementary Table 3.** Mean squares of the analyses of variance restricted to NILs C05 to C08. Inflorescence traits measured at the main spike of each plant.

ns, *,**,***, indicates non-significant and significance at the 0.05, 0.01, and 0.001 probability level, respectively.

Z49: days to awn appearance (flowering time), Z31: days to first node appearance; FLN: final leaf number; Phyll: phyllochron; Grain #: grain number per main spike; Spikelet #: spikelet number per main spike; TGW: thousand grain weight; Grain #.spikelet^-1^: grain number per spikelet.

ns, *,**,***, indicates non-significant and significance at the 0.05, 0.01, and 0.001 probability level, respectively.

**Supplementary Table 4.** Primers sequences used for qRT-PCR. F: forward, R reverse.

| **Target** | **Primer sequence (5'-3')** | **Reference** |
| --- | --- | --- |
| *VRN-H1* | F: TATGAGCGCTACTCTTATGC  R: TGAAGCTCAGAAATGGATTCG | Trevaskis et al*.*, 2006 |
| *VRN-H2* | F: GAGCCACCATCGTGCCATTC  R: GCCGCTTCTTCCTCTTCTC | Trevaskis et al*.*, 2006 |
| *VRN-H3* | F: ATCTCCACTGGTTGGTGACAGA  R: TTGTAGAGCTCGGCAAAGTCC | Yan et al., 2006 |
| *PPD-H1* | F: CAAATCAAAGAGCGGCGATC  R: TCTGACTTGGGATGGTTCACA | Hemming et al*.*, 2008 |
| *PPD-H2* | F: GGTTGTGGCTCATGTTATGC  R: CTACTCCCCTTGAGAACTTTC | Kikuchi et al., 2009  Faure et al., 2007 |
| *ACTIN* | F: GCCGTGCTTTCCCTCTATG  R: GCTTCTCCTTGATGTCCCTTA | Trevaskis et al*.*, 2006 |
| *DCP5* | F: AGCAGAGAAAAATAGACACAGAGAC  R: CCTGTATATCCATACCCTCTTCCATAG | Cantalapiedra et al*.*, 2017 |

**Supplementary Table 5.** Mean squares from the analyses of variance for inflorescence traits measured at the main spike of each plant, corresponding to the eight NILs, including unvernalized C01 and C02 plants, with values equal to zero.

| Source of variation | df | Grain yield  (g) | Grain # | Spikelet # | TGW  (g) | Grain #.  spikelet^-1^ |
| --- | --- | --- | --- | --- | --- | --- |
| **Temperature (T)** | **1** | **9.14 ***** | **3850 ***** | **413.3 ***** | **4942 ***** | **7.61 ***** |
| **Vernalization (V)** | **1** | **0.11 **** | **158 ***** | **552.8 ***** | **1647 **** | **0.33 ***** |
| **Temperature.Vernalization** | **1** | **0.11 *** | **98 ***** | **52.5 ***** | **50 ns** | **0.20 **** |
| **Residual 1** | **12** | **0.01** | **5** | **2.5** | **106** | **0.01** |
| **Genotype (G)** | **7** | **0.32 ***** | **141 ***** | **472.9 ***** | **2953 ***** | **0.48 ***** |
| *VRN-H1* | 1 | 0.33 *** | 288 *** | 1365.0 *** | 468 * | 0.53 *** |
| *VRN-H2* | 1 | 0.25 *** | 95 *** | 288.0 *** | 5860 *** | 0.52 *** |
| *PPD-H1* | 1 | 0.00 ns | 109 *** | 1069.5 *** | 5242 *** | 0.18 *** |
| *VRN-H1*.*VRN-H2* | 1 | 0.62 *** | 215 *** | 450.0 *** | 4051 *** | 1.00 *** |
| *VRN-H1*.*PPD-H1* | 1 | 0.86 *** | 205 *** | 7.0 ns | 4865 *** | 0.99 *** |
| *VRN-H2*.*PPD-H1* | 1 | 0.16 ** | 66 *** | 18.0 ** | 145 ns | 0.11 ** |
| *VRN-H1*.*VRN-H2*.*PPD-H1* | 1 | 0.03 ns | 10 ns | 112.5 *** | 39 ns | 0.02 ns |
| **G*T** | 7 | **0.09 ***** | **103 ***** | **2.9 ns** | **364 **** | **0.18 ***** |
| *VRN-H1*.T | 1 | 0.05 * | 185 *** | 7.0 ns | 41 ns | 0.14 *** |
| *VRN-H2*.T | 1 | 0.24 *** | 50 *** | 0.5 ns | 61 ns | 0.34 *** |
| *PPD-H1*.T | 1 | 0.17 *** | 300 *** | 0.3 ns | 152 ns | 0.23 *** |
| *VRN-H1*.*VRN-H2*.T | 1 | 0.02 ns | 10 ns | 3.1 ns | 148 ns | 0.01 ns |
| *VRN-H1*.*PPD-H1*.T | 1 | 0.07 * | 153 *** | 3.8 ns | 1986 *** | 0.49 *** |
| *VRN-H2*.*PPD-H1*.T | 1 | 0.01 ns | 1 ns | 1.1 ns | 153 ns | 0.01 ns |
| *VRN-H1*.*VRN-H2*.*PPD-H1*.T | 1 | 0.04 ns | 23 * | 4.5 ns | 4 ns | 0.04 ns |
| **G*V** | **7** | **0.39 ***** | **124 ***** | **301.8 ***** | **2408 ***** | **0.31 ***** |
| *VRN-H1*.V | 1 | 0.97 *** | 215 *** | 731.5 *** | 5781 *** | 0.43 *** |
| *VRN-H2*.V | 1 | 0.67 *** | 253 *** | 684.5 *** | 4699 *** | 0.65 *** |
| *PPD-H1*.V | 1 | 0.00 ns | 2 ns | 3.8 ns | 134 ns | 0.00 ns |
| *VRN-H1*.*VRN-H2*.V | 1 | 0.94 *** | 313 *** | 578.0 *** | 5725 *** | 1.07 *** |
| *VRN-H1*.*PPD-H1*.V | 1 | 0.11 ** | 41 ** | 63.3 *** | 69 ns | 0.02 ns |
| *VRN-H2*.*PPD-H1*.V | 1 | 0.02 ns | 7 ns | 6.1 ns | 366 ns | 0.00 ns |
| *VRN-H1*.*VRN-H2*.*PPD-H1*.V | 1 | 0.03 ns | 34 ** | 45.1 *** | 84 ns | 0.00 ns |
| **G*T*V** | **7** | **0.20 ***** | **47 ***** | **5.6 *** | **177 ns** | **0.09 ***** |
| *VRN-H1*.T.V | 1 | 0.78 *** | 190 *** | 22.8 ** | 386 ns | 0.35 *** |
| *VRN-H2*.T.V | 1 | 0.36 *** | 63 *** | 8.0 ns | 447 ns | 0.15 *** |
| *PPD-H1*.T.V | 1 | 0.00 ns | 9 ns | 0.8 ns | 68 ns | 0.02 ns |
| *VRN-H1*.*VRN-H2*.T.V | 1 | 0.14 ** | 43 ** | 3.1 ns | 103 ns | 0.07 * |
| *VRN-H1*.*PPD-H1*.T.V | 1 | 0.01 ns | 9 ns | 0.8 ns | 75 ns | 0.00 ns |
| *VRN-H2*.*PPD-H1*.T.V | 1 | 0.06 * | 8 ns | 0.5 ns | 108 ns | 0.05 * |
| *VRN-H1*.*VRN-H2*.*PPD-H1*.T.V | 1 | 0.04 ns | 5 ns | 3.1 ns | 50 ns | 0.01 ns |
| **Residual 2** | **84** | **0.01** | **4** | **2.4** | **115** | **0.01** |

TGW: thousand grain weight.

ns,*,**,***, indicates non-significant, and significance at the 0.05, 0.01, and 0.001 probability level, respectively.

**Supplementary Table 6.** Mean squares of the analyses of variance of the relative expression of *VRN-H1*, *VRN-H2, VRN-H3,* *PPD-H1* and *PPD-H2*, calculated on mean gene expression at 250 and 350ºC d samples.

| Source of variation |  | | Relative expression | | | | | |
| --- | --- | --- | --- | --- | --- | --- | --- | --- |
|  | df | *VRN-H1* | | *VRN-H2* | *VRN-H3* | | *PPD-H1* | *PPD-H2* |
| **Temperature (T)** | **1** | **0.2 ns** | | **0.12 **** | **0.7 **** | **0.03 *** | | **4.6x10^-3^ ***** |
| **Vernalization (V)** | **1** | **2.7 ***** | | **3.55 ***** | **2.9 ***** | **0.06 **** | | **1.2x10^-4^ ns** |
| **Temperature.Vernalization** | **1** | **0.1 ns** | | **0.14 **** | **0.1 ns** | **0.03 *** | | **7.2x10^-5^ ns** |
| **Residual 1** | **8** | **0.1** | | **0.01** | **1.4** | **0.00** | | **1.8x10^-4^** |
| Sampling time | 1 | 0.3 * | | 0.03 ns | 2.0 *** | 0.02 ns | | 2.9x10^-5^ ns |
| **Genotype (G)** | **7** | **4.0 ***** | | **1.68 ***** | **4.2 ***** | **0.15 ***** | | **2.5x10^-3^ ***** |
| *VRN-H1* | 1 | 6.2 *** | | 3.66 *** | 5.0 *** | 0.22 *** | | 4.6x10^-3^ *** |
| *VRN-H2* | 1 | 0.0 ns | | 3.66 *** | 2.3 *** | 0.09 *** | | 2.3x10^-4^ ns |
| *PPD-H1* | 1 | 1.5 *** | | 0.19 *** | 14.4 *** | 0.58 ***  | | 8.9x10^-3^ *** |
| *VRN-H1*.*VRN-H2* | 1 | 1.6 *** | | 3.66 *** | 2.9 *** | 0.01 ns | | 1.4x10^-5^ ns |
| *VRN-H1*.*PPD-H1* | 1 | 0.0 ns | | 0.19 *** | 2.7 *** | 0.15 *** | | 3.6x10^-3^ *** |
| *VRN-H2*.*PPD-H1* | 1 | 9.0 *** | | 0.19 *** | 1.0 *** | 0.00 ns | | 2.8x10^-4^ ns |
| *VRN-H1*.*VRN-H2.PPD.H1* | 1 | 9.6 *** | | 0.19 *** | 1.0 *** | 0.00 ns | | 4.7x10^-6^ ns |
| **G*T** | **7** | **0.9 ***** | | **0.06 ***** | **0.7 ***** | **0.02 *** | | **1.0x10^-3^ ***** |
| *VRN-H1*.T | 1 | 0.2 * | | 0.12 ** | 0.8 *** | 0.01 ns | | 1.5x10^-3^ ** |
| *VRN-H2*.T | 1 | 2.2 *** | | 0.12 ** | 1.1 *** | 0.00 ns | | 2.2x10^-5^ ns |
| *PPD-H1*.T | 1 | 1.7 *** | | 0.02 ns | 0.4 ** | 0.00 ns | | 4.3x10^-3^ *** |
| *VRN-H1*.*VRN-H2*.T | 1 | 0.2 ns | | 0.12 ** | 0.4 ** | 0.02 ns | | 3.3x10^-4^ ns |
| *VRN-H1*.*PPD-H1*.T | 1 | 1.5 *** | | 0.02 ns | 1.1 *** | 0.01 ns | | 1.3x10^-3^ ** |
| *VRN-H2*.*PPD-H1*.T | 1 | 0.0 ns | | 0.02 ns | 0.9 *** | 0.00 ns | | 3.7x10^-4^ ns |
| *VRN-H1*.*VRN-H2.PPD-H1*.T | 1 | 0.3 * | | 0.02 ns | 0.1 ns | 0.07 ** | | 5.4x10^-6^ ns |
| **G*V** | **7** | **1.1 ***** | | **1.65 ***** | **1.0 ***** | **0.06 ***** | | **2.1x10^-4^ ns** |
| *VRN-H1*.V | 1 | 5.0 *** | | 3.55 *** | 3.5 *** | 0.30 *** | | 6.3x10^-5^ ns |
| *VRN-H2*.V | 1 | 0.1 ns | | 3.55 *** | 0.0 ns | 0.02 ns | | 5.1x10^-5^ ns |
| *PPD-H1*.V | 1 | 0.2 * | | 0.17 *** | 1.2 *** | 0.05 ** | | 7.3x10^-4^ * |
| *VRN-H1*.*VRN-H2*.V | 1 | 0.9 *** | | 3.55 *** | 0.4 ** | 0.00 ns | | 3.4x10^-4^ ns |
| *VRN-H1*.*PPD-H1*.V | 1 | 0.0 ns | | 0.17 *** | 1.3 *** | 0.03 * | | 0.1x10^-6^ ns |
| *VRN-H2*. *PPD-H1*.V | 1 | 0.1 ns | | 0.17 ** | 0.1 ns | 0.00 ns | | 5.4x10^-6^ ns |
| *VRN-H1*.*VRN-H2. PPD-H1*.V | 1 | 1.1 *** | | 0.17 *** | 0.8 *** | 0.04 * | | 2.5x10^-4^ ns |
| **G*T*V** | **7** | **0.0 ns** | | **0.06 ***** | **0.2 *** | **0.01 ns** | | **8.7x10^-4^ ***** |
| *VRN-H1*.T.V | 1 | 0.0 ns | | 0.14 *** | 0.2 * | 0.01 ns | | 1.8x10^-4^ ns |
| *VRN-H2*.T.V | 1 | 0.0 ns | | 0.14 *** | 0.0 ns | 0.05 * | | 1.3x10^-3^ ** |
| *PPD-H1*.T.V | 1 | 0.0 ns | | 0.03 ns | 0.2 * | 0.00 ns | | 3.8x10^-5^ ns |
| *VRN-H1*.*VRN-H2*.T.V | 1 | 0.0 ns | | 0.14 *** | 0.2 * | 0.02 ns | | 1.8x10^-3^ *** |
| *VRN-H1*.*PPD-H1*.T.V | 1 | 0.0 ns | | 0.03 ns | 0.3 * | 0.01 ns | | 2.5x10^-4^ ns |
| *VRN-H2*.*PPD-H1*.T.V | 1 | 0.2 ns | | 0.03 ns | 0.0 ns | 0.00 ns | | 1.1x10^-3^ ** |
| *VRN-H1*.*VRN-H2*.*PPD-H1*.T.V | 1 | 0.1 ns | | 0.03 ns | 0.3 * | 0.00 ns | | 1.5x10^-3^ ** |
| **Residual 2** | **120** | **0.1** | | **0.01** | **0.1** | **0.01** | | **1.4x10^-4^** |

ns, *,**,***, indicates non-significant and significance at the 0.05, 0.01, and 0.001 probability level, respectively.
